# Global seroprevalence of *Leptospira* spp. infection in workers with occupational risk: a systematic review with meta-analysis

**DOI:** 10.3389/fpubh.2026.1931298

**Published:** 2026-10-21

**Authors:** Aksa Ingrid Vieira Batista, Brenna Moizinho Barbosa, Giliene Costa Monteiro Araújo, Tamirys Galvão dos Santos, Audisio Alves da Costa Filho, José Rômulo Soares dos Santos, Roberta Nunes Parentoni, Eloiza Helena Campana, Francisco Bernardino da Silva Neto, Sérgio Santos de Azevedo, Lucas Rannier Ribeiro Antonino Carvalho, Arthur Willian de Lima Brasil

**Affiliations:** 1 Postgraduate Program in Animal Science (PPGCAN), Federal University of Paraíba (UFPB), João Pessoa, Paraíba, Brazil; 2 Molecular Biology Laboratory, Center for Medical Sciences, Federal University of Paraíba (UFPB), João Pessoa, Paraíba, Brazil; 3 Multi-User Laboratory of the Department of Morphology, Center for Health Sciences, Federal University of Paraíba (UFPB), João Pessoa, Paraíba, Brazil; 4 Department of Physiology and Pharmacology, Karolinska Institutet, Stockholm, Sweden

**Keywords:** leptospirosis, occupational risk, prevalence, serodiagnosis, workers

## Abstract

**Introduction:**

Leptospirosis is a globally important zoonosis with a strong occupational component, frequently associated with exposure to environments contaminated by the urine of infected animals. Seroprevalence estimates vary substantially across occupations, geographic regions, and diagnostic approaches. This systematic review and meta-analysis aimed to estimate the global seroprevalence of *Leptospira spp*. among occupationally exposed workers and investigate potential sources of between-study heterogeneity.

**Methods:**

This systematic review and meta-analysis followed PRISMA guidelines and was registered in PROSPERO (CRD420261356167). Searches were conducted in LILACS, MEDLINE, Scopus, Web of Science, PubMed, and ScienceDirect, covering studies published from January 1, 2011 to February 9, 2026. Studies reporting serological diagnosis and quantitative sampling data were included. Methodological quality was assessed using the JBI Critical Appraisal Checklist. Pooled seroprevalence was estimated using a random-effects model, and heterogeneity was assessed using Cochran’s Q and I² statistics. Subgroup analyses were performed according to geographic region, diagnostic method, and occupational category. Sensitivity analyses, funnel plot assessment, Egger’s regression test, and meta-regression were also conducted.

**Results:**

A total of 42 studies comprising 11,155 participants and 2,419 seropositive individuals were included. The pooled global seroprevalence was 18.3% (95% CI: 11.7 -27.4%), with substantial between-study heterogeneity (I² = 96.7%; p < 0.0001). Seroprevalence was higher in Asia and Africa and lower in Europe and the Americas. Geographic region significantly contributed to between-study variability, whereas diagnostic method and occupational category did not significantly explain heterogeneity. Meta-regression revealed a significant decrease in seroprevalence with increasing publication year (β = −0.146, 95% CI: −0.285 to −0.007; χ²M = 4.23, p = 0.0397), explaining 5.81% of the observed heterogeneity. Egger’s regression test indicated funnel plot asymmetry (p = 0.005), suggesting potential small-study effects or publication bias.

**Discussion:**

The findings demonstrate substantial serological evidence of exposure to *Leptospira spp*. among occupationally exposed workers worldwide, with considerable geographic variability and between-study heterogeneity. These results highlight the need for standardized surveillance and diagnostic strategies, as well as targeted occupational health interventions integrating human, animal, and environmental components within a One Health framework.

## 1 Introduction

The One Health approach recognizes the interdependence between human, animal, and environmental health and is particularly relevant to zoonotic diseases such as leptospirosis, which require integrated strategies for prevention and control (1, 2). Leptospirosis is an infectious disease of global importance caused by bacteria of the genus *Leptospira*, considered an occupational zoonosis that affects humans and animals. Its transmission is closely related to contact with water or soil contaminated with the urine of infected animals (3, 4), and in this context, farmers, veterinarians, slaughterhouse workers, and sewage workers exposed to high-risk environments face an increased risk of infection due to work-related factors (5, 6). Therefore, the integrated perspective of the One Health concept emerges as a fundamental component for the prevention, control, and mitigation of leptospirosis impacts.

Recent epidemiological studies have demonstrated substantial variability in serological evidence of *Leptospira* spp. exposure among occupational groups. Studies conducted among sanitation workers, livestock workers, forestry workers, veterinarians, and other occupationally exposed populations have reported markedly different seroprevalence estimates, reflecting differences in occupational activities, environmental exposure, geographic setting, and diagnostic approaches. Recent evidence from sanitation workers, for example, highlights the relevance of occupational exposure in populations with frequent contact with potentially contaminated environments, while studies among forestry and animal-related workers indicate that exposure patterns may vary considerably according to the nature and intensity of occupational contact. These findings suggest that occupational risk is heterogeneous and context-dependent rather than uniformly distributed across professional groups.

Deemed a cosmopolitan zoonosis, leptospirosis particularly affects tropical and subtropical regions due to environmental conditions that favor bacterial survival and transmission between animals and humans (7). It is estimated that more than 500,000 cases of leptospirosis occur annually worldwide, resulting in a significant burden of morbidity and mortality (8). Another relevant consequence is the substantial economic loss caused by leptospirosis due to reduced productivity and its impact on the livestock industry (9).

The diagnosis of leptospirosis can be carried out through direct methods, such as culture and molecular techniques, or indirect methods, with emphasis on serological tests, which are widely used in epidemiological studies and clinical practice. Among these, the microscopic agglutination test (MAT) is considered the gold standard, allowing the detection of antibodies against different serogroups, although it requires specialized laboratory infrastructure and maintenance of live cultures. ELISA stands out for its practicality, suitability for large-scale screening, and good sensitivity, especially for detecting IgM in early stages of infection, while indirect immunofluorescence assay (IFA) presents good sensitivity and specificity, being useful as a complementary method (10–12).

Despite the increasing number of epidemiological studies, evidence regarding occupational exposure to *Leptospira* spp. remains fragmented and difficult to compare across settings. Reported seroprevalence varies substantially among occupational groups and geographic regions, while differences in study populations, exposure profiles, sampling strategies, and serological methods may contribute to this variability. Although recent studies have expanded the evidence base, a comprehensive quantitative synthesis integrating occupationally exposed populations worldwide remains limited. Consequently, the global magnitude of occupational *Leptospira* spp. exposure and the extent to which regional and occupational differences contribute to the observed variation remain insufficiently characterized. Therefore, this systematic review and meta-analysis aimed to estimate the global seroprevalence of *Leptospira* spp. among occupationally exposed workers and to characterize regional and occupational patterns of exposure.

## 2 Methods

### 2.1 Article search strategy and selection

This systematic review and meta-analysis was conducted in accordance with the Preferred Reporting Items for Systematic Reviews and Meta-Analyses (PRISMA) guidelines (13). Published studies related to occupational leptospirosis were retrieved from six electronic databases: LILACS, MEDLINE, Scopus, Web of Science, PubMed, and ScienceDirect. Searches were performed on February 9, 2026, without language restrictions, and included studies published from January 1, 2011 to February 9, 2026.

Search strategies were adapted to the indexing terms and syntax of each database and combined controlled vocabulary and free-text terms related to leptospirosis, occupational exposure, prevalence, and serodiagnosis. Boolean operators (AND and OR) were used to combine the terms according to the search requirements of each database. In PubMed, MeSH terms, including “Leptospirosis” and “Occupational Diseases,” were combined with free-text terms. Database-specific search syntax was applied to Scopus and Web of Science, using TITLE-ABS-KEY and TS fields, respectively. The complete search strategies used in each database are provided in Supplementary Table S1.

All retrieved records were exported in BibTeX and RIS formats to Mendeley® reference manager, where duplicates were removed before independent screening of titles and abstracts. The study protocol was registered in the International Prospective Register of Systematic Reviews (PROSPERO) under registration number CRD420261356167.

### 2.2 Inclusion and exclusion criteria

The eligibility of the articles was assessed by checking for possible discrepancies. For this purpose, inclusion criteria were established for the systematic review and meta-analysis: (1) original peer-reviewed studies with no geographic restriction; (2) studies investigating the seroprevalence of antibodies against *Leptospira* spp. among occupationally exposed individuals; (3) full and accessible texts; (4) reporting total sample size and number of seropositive individuals; and (5) studies using serological diagnostic methods. After rigorous selection, studies that did not meet these criteria, such as those with inconclusive results, indeterminate characteristics, or absence of serological evaluation, were excluded.

### 2.3 Study selection and quality assessment

Study selection was performed independently by two reviewers, who screened the titles and abstracts of all retrieved records and subsequently assessed the full texts of potentially eligible studies. Disagreements between the reviewers were resolved through discussion and consensus. When consensus could not be reached, a third reviewer was consulted to make the final decision.

The methodological quality of the included studies was independently assessed by two reviewers using the Joanna Briggs Institute (JBI) Critical Appraisal Checklist for Studies Reporting Prevalence Data, which comprises nine items (Q1–Q9). Each item was rated as “Yes,” “No,” “Unclear,” or “Not applicable.” The total score was calculated based on the number of “Yes” responses, with higher scores indicating greater methodological quality. Disagreements between reviewers were resolved through discussion and consensus, and a third reviewer was consulted when necessary. The quality assessment was used to characterize the methodological robustness of the included studies and to inform the interpretation of the findings.

### 2.4 Certainty of evidence

The certainty of evidence for the overall pooled seroprevalence and the main subgroup estimates was assessed using an adapted Grading of Recommendations Assessment, Development and Evaluation (GRADE) approach. The assessment considered five domains: risk of bias, inconsistency, indirectness, imprecision, and publication bias. Risk of bias was evaluated based on the JBI Critical Appraisal Checklist assessments of the included studies. Inconsistency was assessed by examining the magnitude of statistical heterogeneity, the overlap of confidence intervals, and the variability of study estimates. Indirectness was evaluated according to the applicability of the populations, occupational exposures, diagnostic methods, and geographic settings to the review question. Imprecision was assessed considering the width of the 95% confidence intervals and the number of studies and participants contributing to each estimate. Publication bias was evaluated through funnel plot inspection and Egger’s regression test when sufficient studies were available. Certainty was classified as high, moderate, low, or very low. The judgments and their justifications are presented in Supplementary Table S3.

### 2.5 Statistical analysis and meta-analysis

A meta-analysis of proportions was performed to estimate the pooled seroprevalence of antibodies against *Leptospira* spp. among occupationally exposed workers. All statistical analyses were conducted exclusively in R version 4.6.1 (R Foundation for Statistical Computing, Vienna, Austria), using the *meta* version 8.5.0 and *metafor* version 5.0.1 packages within RStudio version 2026.07.1 + 147.pro6. All pooled estimates, subgroup analyses, sensitivity and influence analyses, assessments of small-study effects, and meta-regression models reported in this study were generated in R. The complete analysis code and computational session information are provided as Supplementary Table S3 and Supplementary Figure S1 respectively.

Given the expected clinical, methodological, occupational, and geographic variability among studies, pooled prevalence estimates were calculated using a random-effects model. A generalized linear mixed model (GLMM) with a random intercept and logit transformation was fitted, and the between-study variance (τ^2^) was estimated by maximum likelihood. Exact Clopper–Pearson confidence intervals were calculated for the prevalence estimates of individual studies.

In addition to the model-based pooled estimate, a crude aggregated proportion was calculated by dividing the total number of seropositive individuals by the total number of participants across all included studies. This crude proportion was reported descriptively and was not considered a meta-analytic pooled estimate because it does not account for within-study sampling variance or between-study heterogeneity.

Statistical heterogeneity was assessed using Cochran’s Q test and quantified using the I^2^ statistic and τ^2^. I^2^ values of approximately 25%, 50%, and 75% were interpreted as indicating low, moderate, and high heterogeneity, respectively, with values above 75% considered indicative of considerable heterogeneity.

Subgroup analyses were performed according to geographic region (continent), diagnostic method, and occupational category. Diagnostic methods were grouped into microscopic agglutination test (MAT), enzyme-linked immunosorbent assay (ELISA), and ELISA combined with MAT. Occupational exposures were classified into five categories according to similarities in occupational activities and potential exposure pathways: animal-related, agriculture/fishing, sanitation/waste, environmental/mining/forestry, and mixed/other. Differences in pooled prevalence estimates between subgroups were evaluated using the between-subgroup heterogeneity test, denoted as *Q_B_*. Thus, *Q_B_* was used exclusively to represent formal tests comparing the pooled estimates obtained in the stratified subgroup analyses.

Because MAT was the predominant diagnostic method, an additional analysis was restricted to the 28 studies classified as using MAT. The titre used to define seropositivity was identifiable in 24 studies and was categorized as <1:100 or ≥1:100. Studies for which the MAT cut-off could not be determined were classified as “not reported” and were excluded from the comparative subgroup analysis and meta-regression. Pooled seroprevalence estimates for the two cut-off categories were calculated using random-intercept GLMMs, and differences between categories were evaluated using the between-subgroup heterogeneity test χ^2^*_B_*. A univariable mixed-effects meta-regression was subsequently fitted using the cut-off category as a moderator. Effect sizes were expressed on the logit scale, and residual between-study variance was estimated using REML. The overall significance of the moderator was evaluated using χ^2^*_M_*, and the proportion of heterogeneity explained by the cut-off category was quantified using R^2^.

Sensitivity analyses were conducted to evaluate the robustness of the pooled estimate and the influence of individual studies. Influence diagnostics and leave-one-out analyses were performed using random-effects models fitted with restricted maximum likelihood (REML). When a potentially influential study was identified, the primary GLMM was repeated after exclusion of that study to assess the robustness of the pooled estimate. Potential small-study effects and funnel plot asymmetry were evaluated through visual inspection of the funnel plot and Egger’s regression test. Because the negative direction of Egger’s test indicated that smaller studies tended to report lower prevalence estimates, an additional sensitivity analysis was performed by excluding studies in the lowest quartile of sample size. The first quartile corresponded to 152.75 participants; therefore, studies including 152 participants or fewer were excluded. The primary random-intercept GLMM was refitted using the remaining studies, retaining the logit transformation, maximum-likelihood estimation of τ^2^, and Clopper–Pearson confidence intervals for individual-study estimates. The resulting pooled prevalence, 95% confidence interval, and heterogeneity estimates were compared with those of the primary analysis.

Meta-regression analyses were performed to investigate potential sources of between-study heterogeneity. Publication year was initially evaluated as a continuous moderator. Geographic region, diagnostic method, and occupational category were subsequently evaluated as categorical moderators. A multivariable meta-regression model including publication year and geographic region was also fitted to assess whether the temporal association persisted after adjustment for geographic differences. For meta-regression analyses, effect sizes were expressed on the logit scale, and between-study variance was estimated using REML. The overall statistical significance of each moderator was evaluated using the omnibus test of moderators, denoted as *Q_M_*, and the proportion of heterogeneity explained by the moderators was quantified using R^2^. Accordingly, χ^2^*_B_* refers to the test for differences between pooled estimates obtained in stratified subgroup analyses, whereas *Q_M_* refers to the omnibus test of moderator coefficients within the meta-regression model. Because these tests use different model parameterizations and estimation procedures, they are not expected to produce identical numerical values.

Statistical significance was defined as a two-sided *p*-value < 0.05. Because of the substantial between-study heterogeneity expected in prevalence studies, results were interpreted considering pooled estimates, 95% confidence intervals, heterogeneity measures, subgroup analyses, sensitivity analyses, and meta-regression findings rather than statistical significance alone.

## 3 Results

### 3.1 Article selection

The database search identified 1,087 records across six electronic databases. After removal of 501 duplicates, 586 records underwent title and abstract screening, of which 329 were excluded. Following full-text assessment of 257 articles, 215 were excluded, primarily because they lacked serological data, fell outside the predefined study period, or had no available full text. A total of 42 studies met the eligibility criteria and were included in the systematic review and meta-analysis (Figure 1). The included studies generally showed moderate to high methodological quality according to the JBI Critical Appraisal Checklist. Individual scores are presented in Supplementary Table S2.

**Figure 1:**
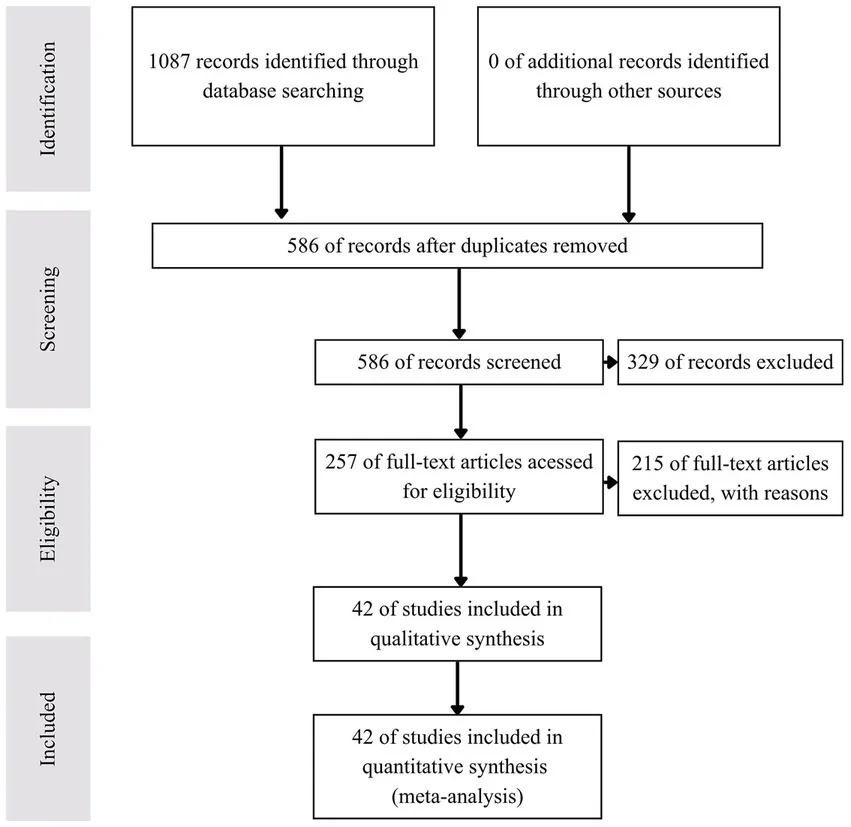
PRISMA flowchart of the study inclusion and exclusion process on the global seroprevalence of *Leptospira* spp. as an occupational disease.

### 3.2 Meta-analysis

The 42 included studies comprised 11,155 occupationally exposed workers, of whom 2,419 were seropositive, corresponding to a crude aggregated proportion of 21.7%. The random-effects generalized linear mixed model yielded a lower pooled global seroprevalence of 18.3% (95% CI: 11.7–27.4%). This difference is expected because the crude proportion is driven by the total number of participants and gives greater influence to larger studies, whereas the random-effects GLMM combines study-specific prevalence estimates on the logit scale while accounting for within-study sampling variance and between-study heterogeneity. Considerable between-study heterogeneity was observed (τ^2^ = 2.8823; I^2^ = 96.7%, 95% CI: 96.1–97.2%; χ^2^ = 1,224.98, df = 41, *p* < 0.0001), indicating substantial variability in prevalence estimates across studies (Figures 2, 3).

**Figure 2:**
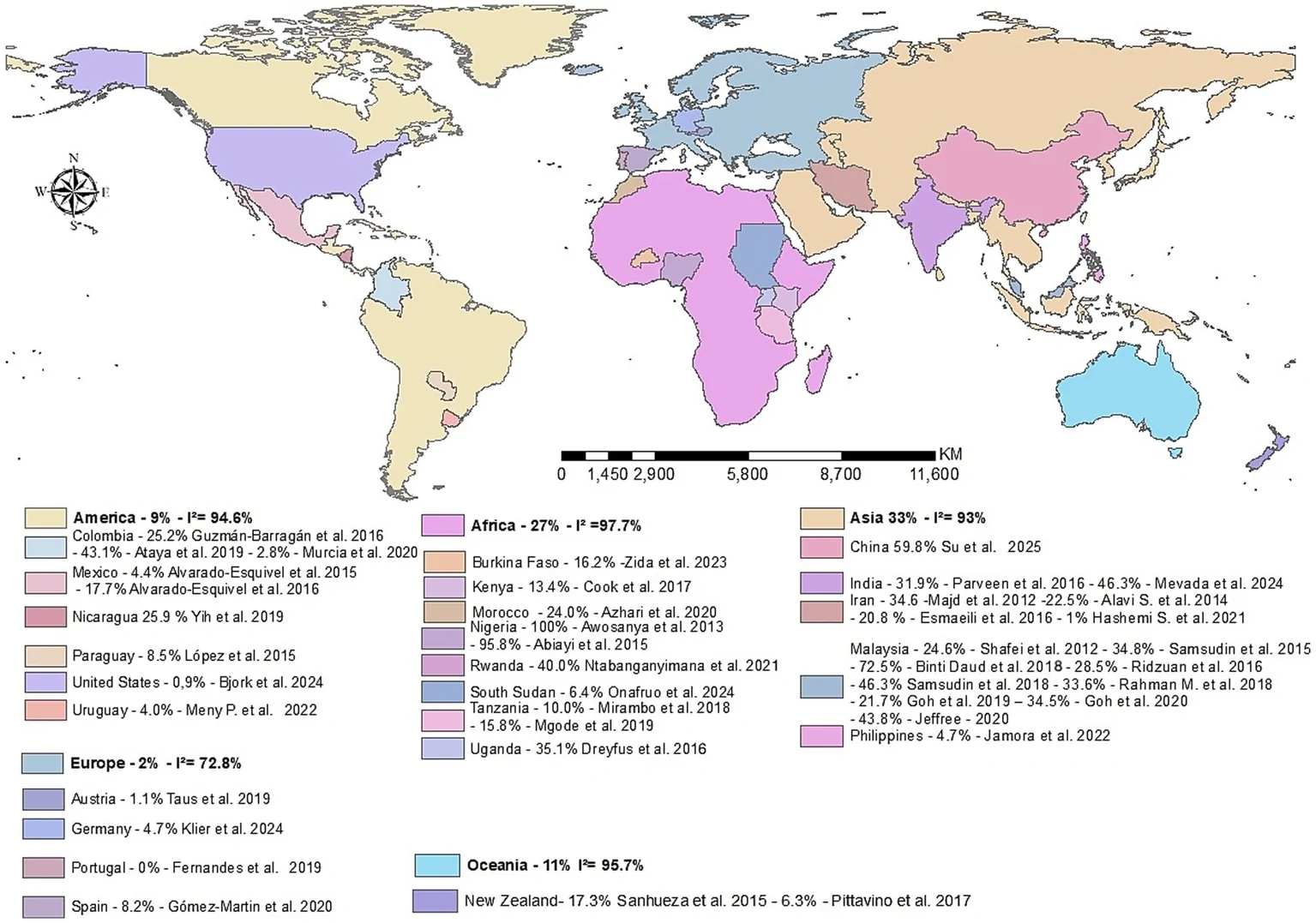
Global seroprevalence of *Leptospira* spp. as an occupational disease.

**Figure 3:**
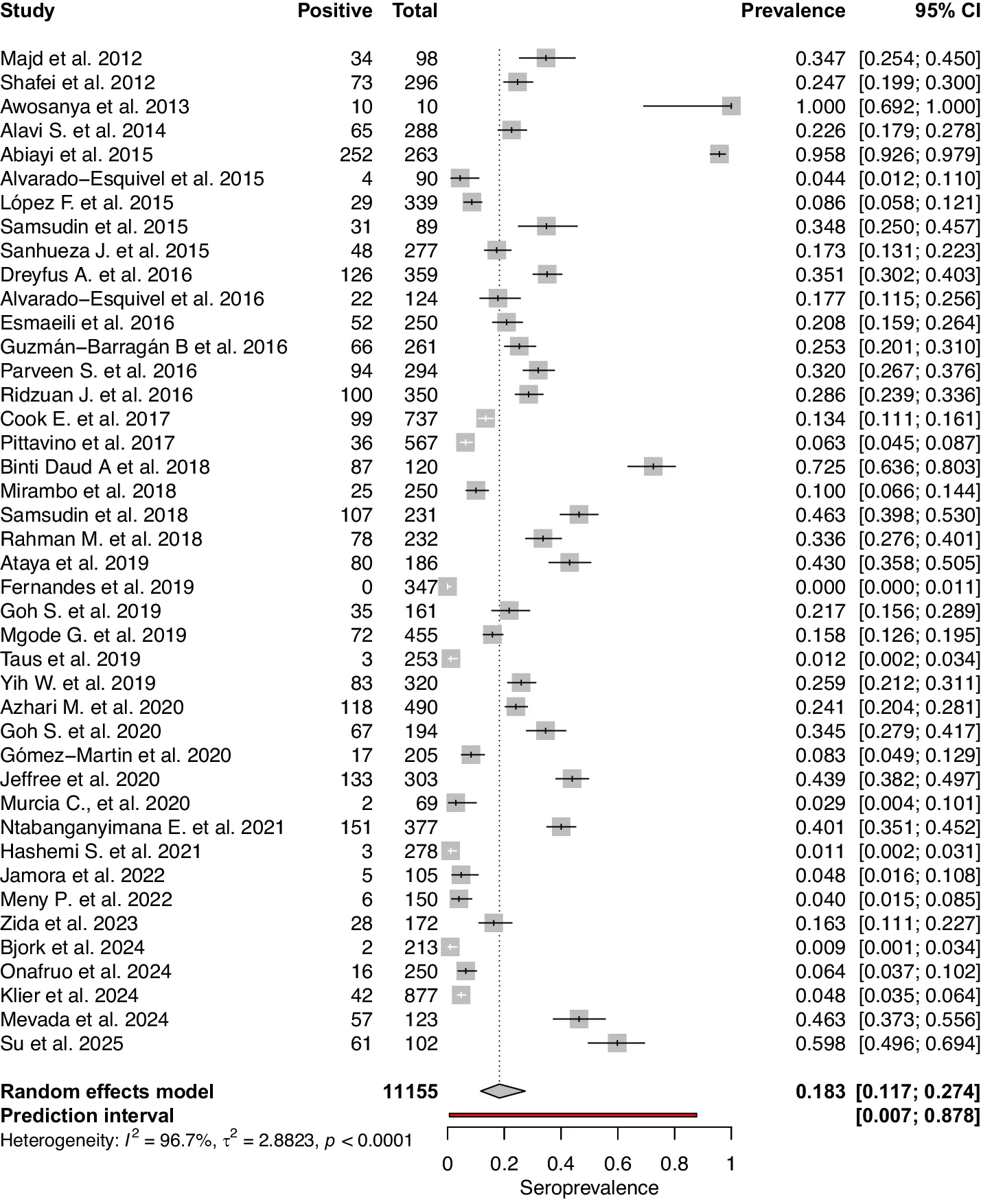
Forest plot of the global seroprevalence of antibodies against *Leptospira* spp. among occupationally exposed workers in studies published between 1 January 2011 and 9 February 2026. The analysis included 42 studies comprising 11,155 participants. Study-specific prevalence estimates and exact Clopper–Pearson 95% confidence intervals are represented by squares and horizontal lines, respectively. The pooled estimate was obtained using a random-effects random-intercept generalized linear mixed model with logit transformation and maximum-likelihood estimation of between-study variance. Estimates were back-transformed from the logit scale and are presented as proportions. The diamond represents the pooled estimate and its 95% confidence interval.

Of the 42 included studies, 28 were classified as using MAT, nine as using ELISA, and five as using ELISA combined with MAT for the serological outcome included in the meta-analysis.

Occupational exposures were classified into five categories according to similarities in occupational activities and potential exposure pathways to *Leptospira* spp. The animal-related category included 22 studies involving slaughterhouse workers, meat workers, veterinarians, livestock workers, animal handlers, kennel workers, dog handlers, and other occupations involving direct contact with animals or animal-derived materials. The sanitation/waste category included eight studies involving waste collection, sewage, sanitation, and related activities. Agriculture/fishing comprised four studies involving agricultural, plantation, fishing, or aquatic-resource activities. Environmental/mining/forestry comprised three studies involving forestry, mining, and predominantly environmental exposures. Five studies involving heterogeneous occupational populations that could not be assigned to a single predominant exposure category were classified as mixed/other (Table 1).

**Table 1:** Characteristics, diagnostic methods, MAT cut-off values, and methodological quality of studies included in the meta-analysis of occupational exposure to *Leptospira* spp.

Study	Positive	Total	Prevalence	Country	Continent	Diagnostic test	Occupation group	JBI score	Quality	MAT cut-off	MAT cut-off group
Majd et al. (26)	34	98	34.7%	Iran	Asia	MAT	Animal-related	8	High	200	≥1:100
Awosanya et al. (18)	10	10	100.0%	Nigeria	Africa	ELISA + MAT	Animal-related	7	High	NR	NR
Shafei et al. (27)	73	296	24.7%	Malaysia	Asia	MAT	Sanitation/Waste	9	High	100	≥1:100
Alavi et al. (28)	65	288	22.6%	Iran	Asia	ELISA	Agriculture/Fishing	9	High	–	–
Abiayi et al. (14)	252	263	95.8%	Nigeria	Africa	MAT	Animal-related	8	High	NR	NR
Alvarado-Esquivel et al. (29)	4	90	4.4%	Mexico	Americas	ELISA	Sanitation/Waste	8	High	–	–
López et al. (30)	29	339	8.6%	Paraguay	Americas	ELISA	Sanitation/Waste	9	High	–	–
Samsudin et al. (31)	31	89	34.8%	Malaysia	Asia	MAT	Sanitation/Waste	8	High	50	<1:100
Sanhueza et al. (32)	48	277	17.3%	New Zealand	Oceania	MAT	Animal-related	8	High	48	<1:100
Alvarado-Esquivel et al. (33)	22	124	17.7%	Mexico	Americas	ELISA	Animal-related	9	High	–	–
Dreyfus et al. (34)	126	359	35.1%	Uganda	Africa	MAT	Animal-related	8	High	48	<1:100
Esmaeili et al. (35)	52	250	20.8%	Iran	Asia	ELISA	Animal-related	8	High	–	–
Guzmán-Barragán et al. (36)	66	261	25.3%	Colombia	Americas	ELISA + MAT	Mixed/Other	8	High	100	≥1:100
Parveen et al. (37)	94	294	32.0%	India	Asia	MAT	Environmental/Mining/Forestry	9	High	20	<1:100
Ridzuan et al. (22)	100	350	28.6%	Malaysia	Asia	MAT	Agriculture/Fishing	8	High	100	≥1:100
Cook et al. (38)	99	737	13.4%	Kenya	Africa	ELISA	Animal-related	9	High	–	–
Pittavino et al. (39)	36	567	6.3%	New Zealand	Oceania	MAT	Animal-related	8	High	24	<1:100
Daud et al. (16)	87	120	72.5%	Malaysia	Asia	MAT	Animal-related	9	High	100	≥1:100
Mirambo et al. (40)	25	250	10.0%	Tanzania	Africa	MAT	Animal-related	9	High	80	<1:100
Samsudin et al. (41)	107	231	46.3%	Malaysia	Asia	MAT	Mixed/Other	9	High	100	≥1:100
Rahman et al. (42)	78	232	33.6%	Malaysia	Asia	MAT	Mixed/Other	9	High	100	≥1:100
Ataya et al. (43)	80	186	43.0%	Colombia	Americas	MAT	Animal-related	9	High	NR	NR
Fernandes et al. (44)	0	347	0.0%	Portugal	Europe	MAT	Sanitation/Waste	9	High	NR	NR
Goh et al. (19)	35	161	21.7%	Malaysia	Asia	MAT	Animal-related	8	High	50	<1:100
Mgode et al. (45)	72	455	15.8%	Tanzania	Africa	MAT	Agriculture/Fishing	9	High	20	<1:100
Taus et al. (46)	3	253	1.2%	Austria	Europe	MAT	Animal-related	9	High	100	≥1:100
Yih et al. (47)	83	320	25.9%	Nicaragua	Americas	MAT	Environmental/Mining/Forestry	8	High	100	≥1:100
Azhari et al. (20)	118	490	24.1%	Morocco	Africa	ELISA + MAT	Animal-related	9	High	50	<1:100
Goh et al. (48)	67	194	34.5%	Malaysia	Asia	MAT	Animal-related	9	High	50	<1:100
Gómez-Martín et al. (49)	17	205	8.3%	Spain	Europe	ELISA	Agriculture/Fishing	9	High	–	–
Jeffree et al. (50)	133	303	43.9%	Malaysia	Asia	MAT	Sanitation/Waste	8	High	50	<1:100
Murcia et al. (51)	2	69	2.9%	Colombia	Americas	MAT	Animal-related	8	High	100	≥1:100
Hashemi et al. (15)	3	278	1.1%	Iran	Asia	MAT	Mixed/Other	8	High	100	≥1:100
Ntabanganyimana et al. (52)	151	377	40.1%	Rwanda	Africa	ELISA + MAT	Mixed/Other	8	High	100	≥1:100
Jamora et al. (17)	5	105	4.8%	Philippines	Asia	MAT	Sanitation/Waste	8	High	100	≥1:100
Meny et al. (53)	6	150	4.0%	Uruguay	Americas	MAT	Animal-related	8	High	200	≥1:100
Zida et al. (54)	28	172	16.3%	Burkina Faso	Africa	ELISA + MAT	Animal-related	8	High	50	<1:100
Bjork et al. (21)	2	213	0.9%	United States	Americas	MAT	Animal-related	8	High	100	≥1:100
Klier et al. (55)	42	877	4.8%	Germany	Europe	ELISA	Environmental/Mining/Forestry	8	High	–	–
Onafruo et al. (56)	16	250	6.4%	South Sudan	Africa	MAT	Animal-related	9	High	100	≥1:100
Mevada et al. (57)	57	123	46.3%	India	Asia	ELISA + MAT	Animal-related	9	High	50	<1:100
Su et al. (4)	61	102	59.8%	China	Asia	ELISA	Sanitation/Waste	8	High	–	–

NR, not reported; MAT, microscopic agglutination test; ELISA, enzyme-linked immunosorbent assay.

Statistically significant occupational, environmental, behavioral, and demographic associations reported in the included studies varied across geographic regions and occupational settings (Table 2). These factors encompassed demographic characteristics, occupational activities, contact with animals or potentially contaminated biological materials, environmental exposures, and work-related practices. Given the heterogeneity in the definition, measurement, and statistical assessment of risk factors across studies, these findings were summarized descriptively and were not interpreted as measures of the frequency or relative epidemiological importance of individual risk factors.

**Table 2:** Quantitative associations between occupational, environmental, and behavioral factors and *Leptospira* seropositivity reported in the included studies.

Continent	Study	Factor/exposure	Effect measure	Estimate (95% CI)	*p*-value
Africa	Abiayi et al. (14)	Age 18–25 years	OR	7.40 (1.47–50.63)	<0.05
	Awosanya et al. (18)	Wounds or cuts on hands/legs	OR	12.0 (1.7–147.5)	0.01
	Awosanya et al. (18)	Contact with kennel sewage	OR	15.0 (1.5–759)	0.01
	Dreyfus et al. (34)	Skinning cattle	OR	12.3 (1.4–108.6)	0.024
	Dreyfus et al. (34)	Living in close proximity to monkeys	OR	1.92 (1.2–3.1)	0.009
	Cook et al. (38)	Cleaning offal	OR	5.1 (1.8–15.0)	0.003
	Cook et al. (38)	Having a wound at interview	OR	3.1 (1.5–6.1)	0.001
	Cook et al. (38)	Smoking	OR	1.8 (1.1–2.9)	—
	Cook et al. (38)	Eating at the slaughterhouse	OR	2.1 (1.2–3.6)	0.010
	Cook et al. (38)	Borehole for personal water use	OR	2.3 (1.1–4.7)	—
	Cook et al. (38)	Slaughterhouse with a roof	OR	2.6 (1.2–5.6)	0.015
	Cook et al. (38)	Well/spring water at slaughterhouse	OR	2.2 (1.2–4.0)	0.007
	Cook et al. (38)	Wearing protective aprons	OR	0.4 (0.2–0.7)	0.001
	Ntabanganyimana et al. (52)	Slaughterhouse worker	OR	2.409 (1.061–5.469)	0.036
	Ntabanganyimana et al. (52)	Livestock farmer	OR	4.021 (1.636–9.886)	0.002
	Ntabanganyimana et al. (52)	Cattle farmer	OR	4.727 (1.863–11.997)	0.001
	Zida et al. (54)	Working at bleeding station	OR	5.51 (1.19–29.5)	0.03
	Zida et al. (54)	Contact with feces and urine	OR	2.50 (0.97–6.65)	0.04†
	Zida et al. (54)	Agriculture outside the slaughterhouse	OR	4.28 (0.79–20.80)	0.05†
	Onafruo et al. (56)	Flaying	aOR	14.9 (2.5–88.9)	0.003
	Onafruo et al. (56)	Wearing aprons/overalls	aOR	10.6 (1.6–67.6)	0.012
	Onafruo et al. (56)	Number of carcasses handled per day*	aOR	2.7 (1.6–4.5)	0.001
Americas	Alvarado-Esquivel et al. (33)	Duration in meat-working activity >10 years	OR	3.87 (1.13–13.22)	0.03
	Alvarado-Esquivel et al. (33)	Rural residence	OR	4.29 (1.29–14.20)	0.01
	Alvarado-Esquivel et al. (33)	Snake meat consumption	OR	7.20 (1.48–34.91)	0.01
	Alvarado-Esquivel et al. (33)	Consumption of unwashed raw fruits	OR	6.68 (1.16–38.55)	0.03
	Guzmán-Barragán et al. (36)	Work in animal slaughter/processing plants	OR	1.86 (1.02–3.38)	0.019
Asia	Alavi et al. (28)	Male sex among rice farmers	OR	2.16 (1.05–4.48)	0.05
	Alavi et al. (28)	Age >35 years among rice farmers	OR	2.18 (1.04–4.56)	0.03
	Ridzuan et al. (22)	Cows present in plantation	aOR	4.78 (2.76–8.26)	<0.05
	Ridzuan et al. (22)	Landfill site in plantation	aOR	2.04 (1.22–3.40)	<0.05
	Daud et al. (16)	Pathogenic *Leptospira* detected in farm environment	aOR	4.15 (1.15–14.99)	<0.05
	Daud et al. (16)	Garbage dumping in farm	aOR	2.40 (1.02–5.65)	<0.05
	Su et al. (4)	Long working hours	OR	1.92 (1.22–3.02)	0.004
	Su et al. (4)	Working in garbage disposal	OR	1.68 (1.05–2.70)	0.03
	Su et al. (4)	Use of mask	OR	0.55 (0.32–0.94)	0.03
	Su et al. (4)	Use of boots	OR	0.33 (0.18–0.61)	<0.001
	Su et al. (4)	Cleaning with soap	OR	0.43 (0.23–0.79)	0.007
Europe	Gómez-Martín et al. (49)	Red swamp crayfish collecting	OR	3.40 (1.03–11.17)	0.044
	Gómez-Martín et al. (49)	Male sex	OR	13.18 (1.72–101.12)	0.013
	Klier et al. (55)	Canoeing	aOR	2.35 (1.02–5.42)	0.044
	Klier et al. (55)	Touching rodents	aOR	2.40 (1.14–4.97)	0.021
	Klier et al. (55)	Frequently cleaning animal cages/stables	aOR	0.20 (0.074–0.54)	0.002
Oceania	Sanhueza et al. (32)	Home slaughter of cattle	OR	4.6 (1.3–16.1)	0.03
	Sanhueza et al. (32)	Home slaughter of pigs	OR	7.9 (1.7–37.5)	0.02
	Sanhueza et al. (32)	Mid-high occupational exposure to dogs/cats	OR	9.2 (1.4–62.8)	—
	Pittavino et al. (39)	Offal removal/pet food work	OR	22.1 (2.3–209.8)	0.001
	Pittavino et al. (39)	Intestine/kidney removal or meat inspection	OR	33.8 (4.2–271.1)	<0.001
	Pittavino et al. (39)	Yard work, stunning or pelting	OR	57.0 (6.9–473.3)	<0.001

OR, odds ratio; aOR, adjusted odds ratio; CI, confidence interval. Values are presented as reported in the original studies. Estimates <1 indicate protective associations. *For Onafruo et al., the estimate represents the increase in odds for each additional carcass handled per day. †For Zida et al. (54) the *p*-values and 95% CIs are reproduced exactly as reported by the authors; the published *p*-values indicate statistical significance although the reported confidence intervals include 1.0.

### 3.3 Global and regional patterns of *Leptospira* spp. seroprevalence

Significant differences in pooled seroprevalence were observed across continents (χ^2^ = 27.73, df = 4, *p* < 0.0001). The pooled estimates were 33% (95% CI: 25–42%) in Asia, 27% (95% CI: 9–58%) in Africa, 11% (95% CI: 5–21%) in Oceania, 9% (95% CI: 4–20%) in the Americas, and 2% (95% CI: 0–9%) in Europe (Figure 4). However, the estimates for Africa and Europe should be interpreted cautiously because of their wide confidence intervals and, particularly for Europe, the small number of contributing studies (*n* = 4), which resulted in limited precision. Considerable residual heterogeneity remained within Asia (I^2^ = 93.0%), Africa (I^2^ = 97.7%), the Americas (I^2^ = 94.6%), and Oceania (I^2^ = 95.7%), while heterogeneity was lower, although still substantial, in Europe (I^2^ = 72.8%).

**Figure 4:**
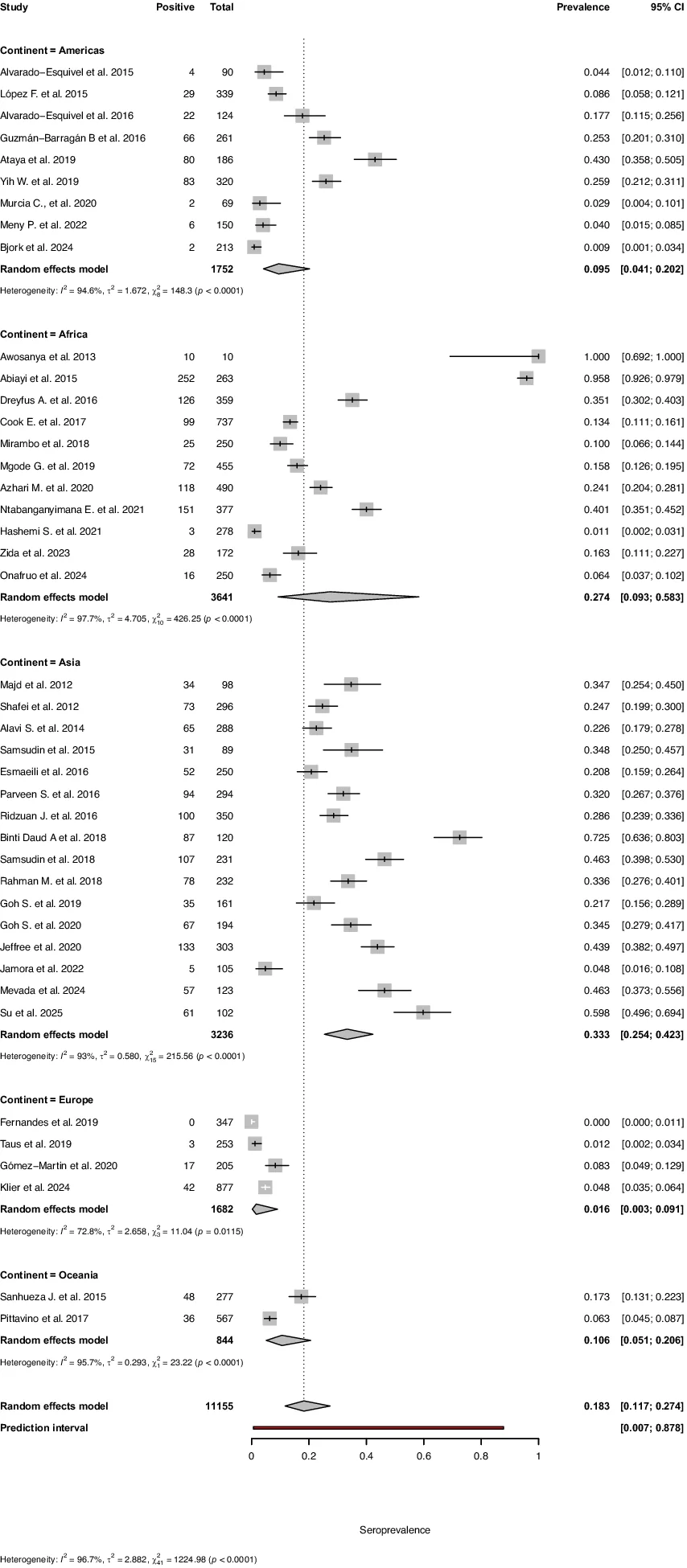
Forest plot of occupational *Leptospira* spp. seroprevalence stratified by geographic region. The analysis included 42 studies distributed across Asia, Africa, the Americas, Europe, and Oceania. Study-specific prevalence estimates and exact Clopper–Pearson 95% confidence intervals are represented by squares and horizontal lines, respectively. Random-effects subgroup estimates were obtained using random-intercept generalized linear mixed models with logit transformation and maximum-likelihood estimation of between-study variance. Estimates were back-transformed from the logit scale and are presented as proportions. Diamonds represent subgroup-specific pooled estimates and their 95% confidence intervals. Differences between continents were evaluated using χ^2^*_B_*.

### 3.4 Subgroup analysis by diagnostic method

Pooled seroprevalence was 18% (95% CI: 9–32%) among studies using MAT, 14% (95% CI: 8–24%) among studies using ELISA, and 29% (95% CI: 20–40%) among studies using ELISA combined with MAT. Although the pooled estimate was numerically higher among studies using ELISA combined with MAT, the formal test for subgroup differences did not reach statistical significance (χ^2^ = 5.71, df = 2, *p* = 0.0575). Considerable residual heterogeneity remained within all diagnostic subgroups (I^2^ = 93.1–96.5%) (Figure 5).

**Figure 5:**
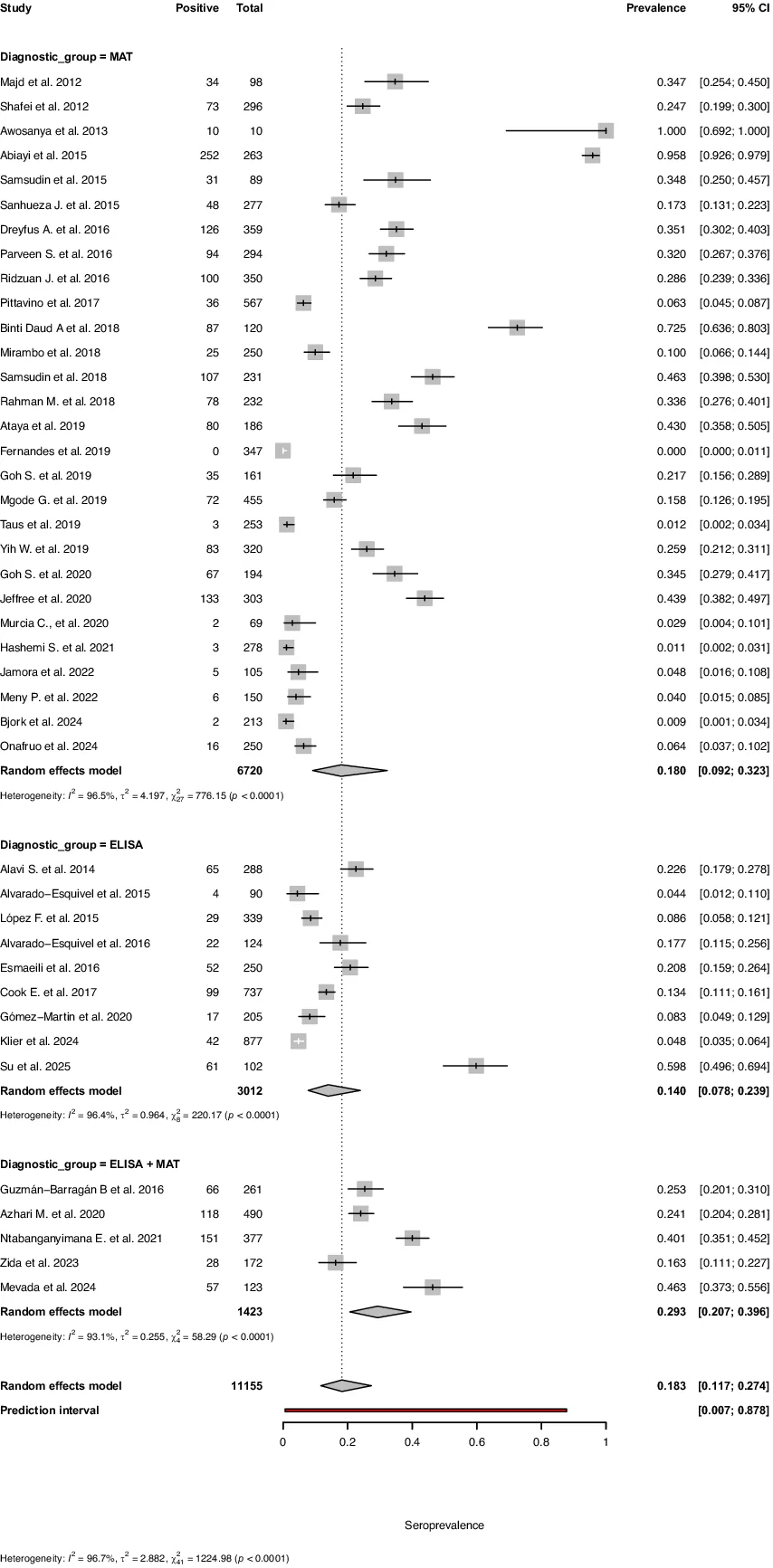
Forest plot of occupational *Leptospira* spp. seroprevalence stratified by serological diagnostic method. The analysis included 42 studies: 28 classified as MAT, nine as ELISA, and five as ELISA combined with MAT. Study-specific prevalence estimates and exact Clopper–Pearson 95% confidence intervals are represented by squares and horizontal lines, respectively. Random-effects subgroup estimates were obtained using random-intercept generalized linear mixed models with logit transformation and maximum-likelihood estimation of between-study variance. Estimates were back-transformed from the logit scale and are presented as proportions. Diamonds represent subgroup-specific pooled estimates and their 95% confidence intervals. Differences between diagnostic methods were evaluated using χ^2^*_B_*.

### 3.5 Subgroup analysis by occupational category

Pooled seroprevalence was 20.9% (95% CI: 10.7–36.9%) for animal-related occupations, 11.4% (95% CI: 2.9–35.7%) for sanitation/waste workers, 17.8% (95% CI: 11.3–26.8%) for agriculture/fishing workers, 21.0% (95% CI: 6.4–50.9%) for mixed/other occupational exposures, and 16.8% (95% CI: 6.2–38.3%) for environmental/mining/forestry workers. No significant differences were detected between occupational categories (χ^2^ = 0.84, df = 4, *p* = 0.9334) (Figure 6). Consistently, occupational category was not a significant moderator in the meta-regression (χ^2^*_M_* = 0.50, *p* = 0.9738; R^2^ = 0%), indicating that the occupational grouping did not explain a measurable proportion of the between-study heterogeneity.

**Figure 6:**
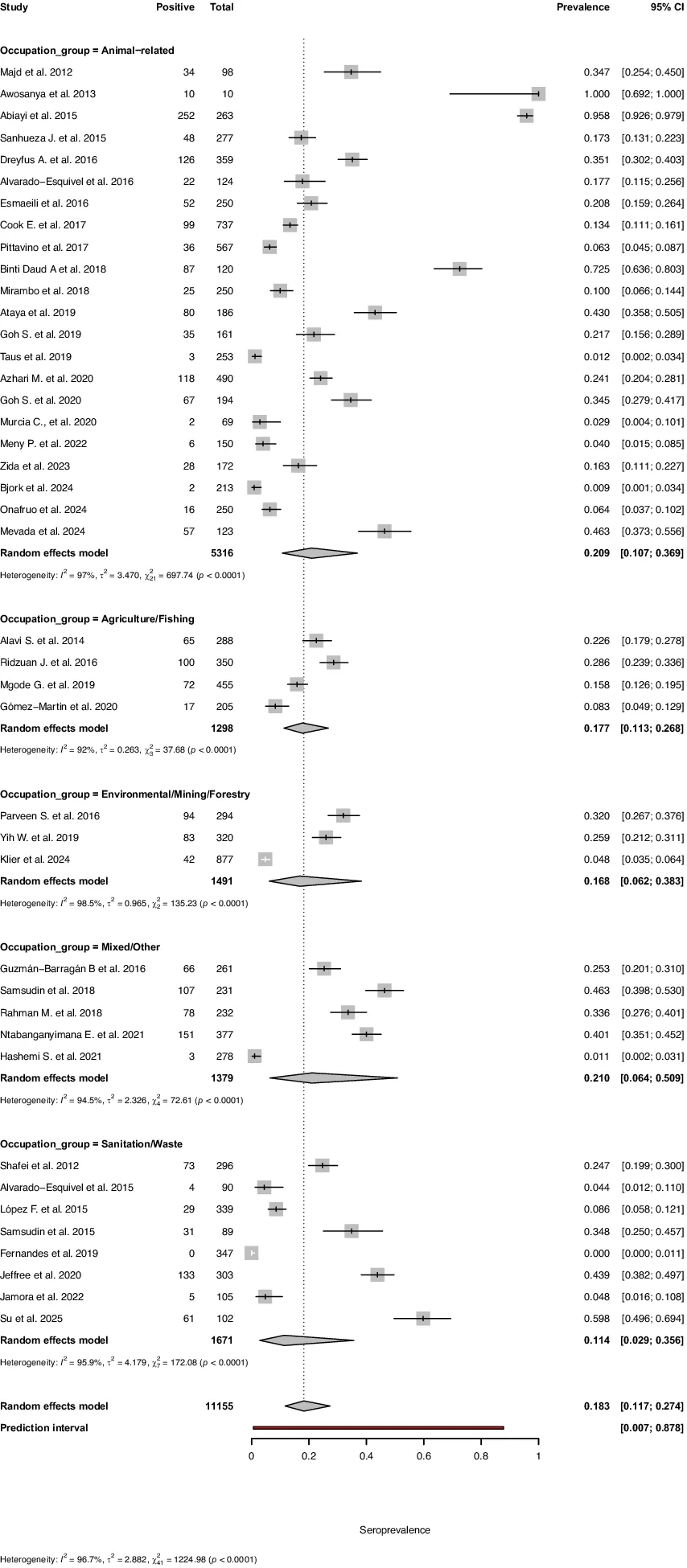
Forest plot of occupational *Leptospira* spp. seroprevalence stratified by occupational category. The analysis included 42 studies classified as animal-related, sanitation/waste, agriculture/fishing, environmental/mining/forestry, or mixed/other occupational exposures. Study-specific prevalence estimates and exact Clopper–Pearson 95% confidence intervals are represented by squares and horizontal lines, respectively. Random-effects subgroup estimates were obtained using random-intercept generalized linear mixed models with logit transformation and maximum-likelihood estimation of between-study variance. Estimates were back-transformed from the logit scale and are presented as proportions. Diamonds represent subgroup-specific pooled estimates and their 95% confidence intervals. Differences between occupational categories were evaluated using χ^2^*_B_.*

### 3.6 Sensitivity and influence analyses

Leave-one-out sensitivity analysis demonstrated that exclusion of individual studies did not materially alter the pooled prevalence estimate. Influence diagnostics identified Abiayi et al. (14) as a potentially influential study. Exclusion of this study reduced the pooled prevalence from 18.3% (95% CI: 11.7–27.4%) to 16.7% (95% CI: 11.1–24.4%), while considerable heterogeneity persisted (I^2^ = 96.1%). These findings indicate that the overall pooled estimate was not driven by a single study.

Targeted sensitivity analyses were conducted for the three studies presenting extreme observed proportions: Awosanya et al. (18) (10/10), Abiayi et al. (14) (252/263), and Fernandes et al. (44) (0/347). Excluding Awosanya et al. yielded a pooled seroprevalence of 17.0% (95% CI: 11.1–25.2%), whereas exclusion of Abiayi et al. (14) yielded an estimate of 16.7% (95% CI: 11.1–24.4%). After exclusion of Fernandes et al., the pooled estimate was 19.9% (95% CI: 13.3–28.8%). Simultaneous exclusion of all three studies resulted in a pooled seroprevalence of 17.1% (95% CI: 12.1–23.6%), compared with 18.3% (95% CI: 11.7–27.4%) in the primary analysis. The corresponding between-study variance decreased from τ^2^ = 2.8823 to τ^2^ = 1.5887. Thus, although these studies contributed to between-study heterogeneity, their exclusion did not materially alter the pooled estimate.

### 3.7 Funnel plot asymmetry and small-study effects

Egger’s regression test indicated statistically significant funnel plot asymmetry (z = −2.79, *p* = 0.0053). Importantly, the negative direction of the test indicated that smaller studies tended to report lower, rather than higher, seroprevalence estimates. Thus, the observed asymmetry was opposite to the conventional pattern associated with preferential publication of small studies reporting high prevalence estimates. Accordingly, this finding was interpreted as evidence of potential small-study effects rather than evidence that publication bias had inflated the pooled seroprevalence.

In the sensitivity analysis, the five studies with the smallest sample sizes were excluded, leaving 37 studies comprising 10,799 participants and 2,338 seropositive individuals. After exclusion, the pooled seroprevalence was 17.5% (95% CI: 11.1–26.5%), compared with 18.3% (95% CI: 11.7–27.4%) in the primary analysis. Considerable heterogeneity persisted (I^2^ = 97.0%; τ^2^ = 2.6266). The modest change in the pooled estimate and the substantial overlap between confidence intervals indicated that the overall result was robust to the exclusion of the smallest studies.

Funnel plot asymmetry remained statistically significant after the simultaneous exclusion of Awosanya et al. (18), Abiayi et al. (14), and Fernandes et al. (44) (Egger’s regression test: t = −2.92, df = 37, *p* = 0.0059). Therefore, the asymmetry observed in the primary analysis was not explained solely by these three studies.

### 3.8 Meta-regression

Visual inspection of the funnel plot suggested asymmetry, which was supported by Egger’s regression test (z = −2.79, *p* = 0.0053) (Figure 7). Given the considerable between-study heterogeneity, this finding was interpreted cautiously as evidence of potential small-study effects rather than definitive evidence of publication bias.

**Figure 7:**
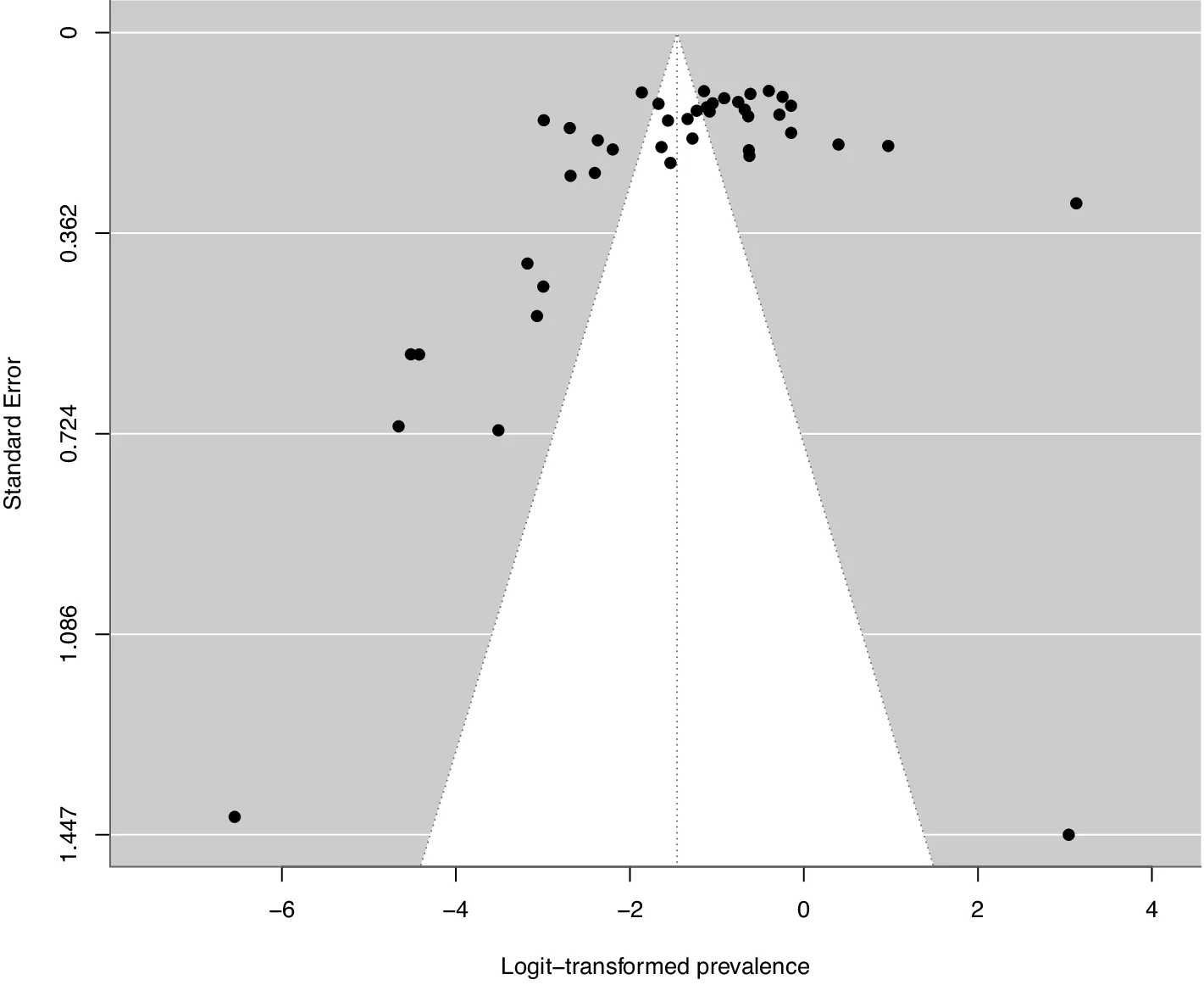
Funnel plot for assessment of small-study effects in the meta-analysis of occupational *Leptospira* spp. seroprevalence.

In univariable meta-regression, publication year was negatively associated with seroprevalence (*β* = −0.146, 95% CI: −0.285 to −0.007; χ^2^*_M_* = 4.23, *p* = 0.0397), accounting for 5.81% of between-study heterogeneity. Continent was also a significant moderator (χ^2^*_M_* = 18.09, *p* = 0.0012) and accounted for 25.68% of heterogeneity. In contrast, diagnostic method (*Q_M_* = 1.12, *p* = 0.5700; R2 = 0%) and occupational category (χ^2^*_M_* = 0.50, *p* = 0.9738; R2 = 0%) were not significant moderators. Although some individual studies reported more detailed exposure-related variables, including duration of occupational activity, working hours, protective clothing, and specific occupational tasks, these variables were not sufficiently standardized across studies to permit quantitative moderator analysis.

In the multivariable model including publication year and continent, the overall moderator test remained significant (χ^2^*_M_* = 21.07, *p* = 0.0008), and the model accounted for 27.30% of between-study heterogeneity. After adjustment for continent, the association with publication year was attenuated and was no longer statistically significant (*β* = −0.108, 95% CI: −0.237 to 0.022; *p* = 0.1023). Compared with Asia, seroprevalence remained significantly lower in the Americas (*β* = −1.439, 95% CI: −2.546 to −0.331; *p* = 0.0109) and Europe (*β* = −2.664, 95% CI: −4.278 to −1.049; *p* = 0.0012). No significant differences were observed for Africa (*β* = −0.289, 95% CI: −1.339 to 0.761; *p* = 0.5896) or Oceania (*β* = −1.619, 95% CI: −3.575 to 0.336; *p* = 0.1046). Considerable residual heterogeneity persisted (χ^2^*_E_* = 820.51, *p* < 0.0001; I2 = 98.30%; τ2 = 1.7162). The univariable temporal association is illustrated in Figure 8.

**Figure 8:**
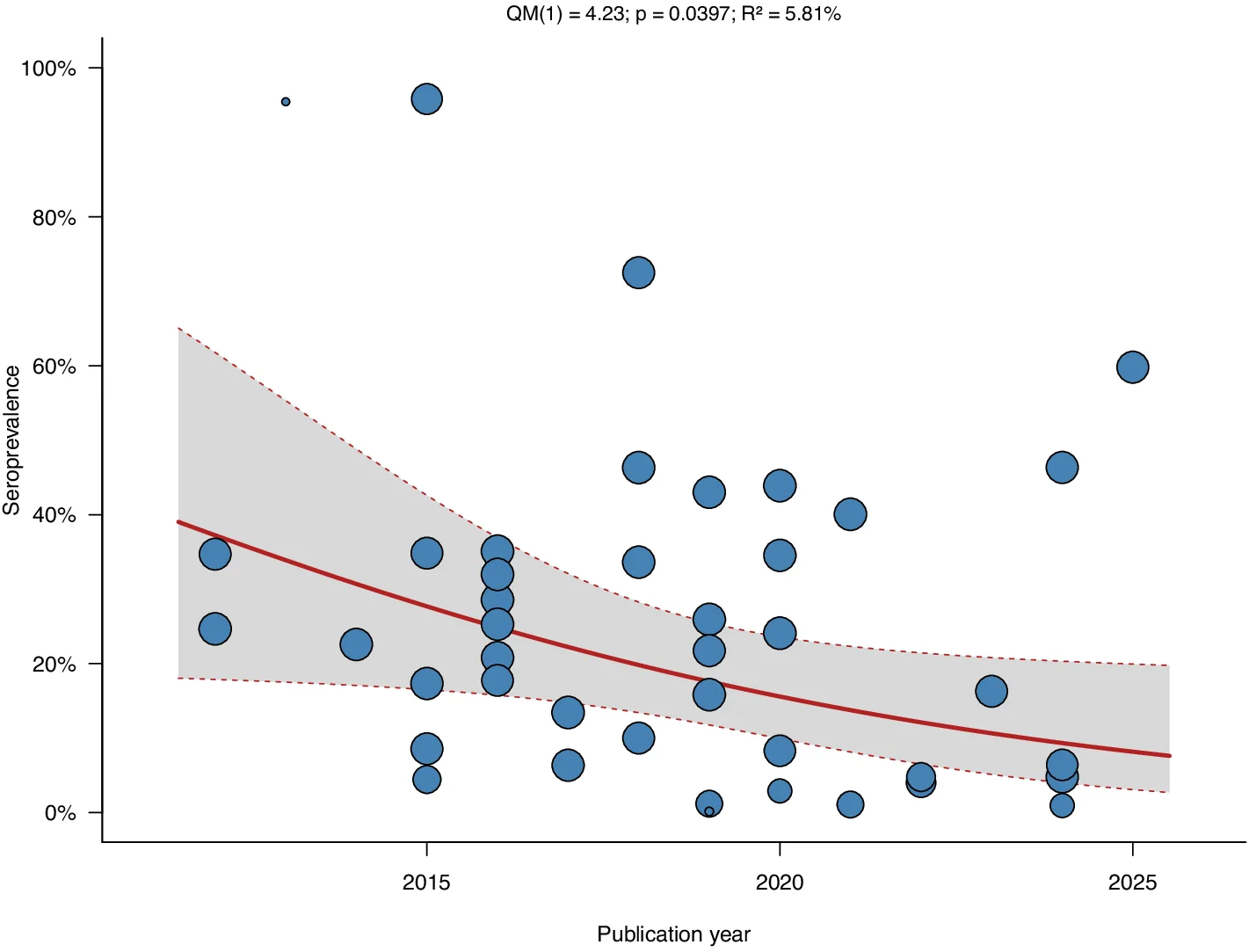
Bubble plot of the univariable meta-regression between publication year and *Leptospira* spp. seroprevalence.

### 3.9 MAT cut-off subgroup analysis and meta-regression

Among the 28 studies classified as using MAT, the cut-off titre used to define seropositivity was identifiable in 24 studies. Ten studies used titres below 1:100, 14 used titres of 1:100 or higher, and four did not provide sufficient information to determine the cut-off.

In the subgroup analysis, studies using MAT cut-offs below 1:100 had a pooled seroprevalence of 22.6% (95% CI: 15.5–31.8%), whereas studies using cut-offs of 1:100 or higher had a pooled seroprevalence of 11.0% (95% CI: 4.6–24.0%). Although the pooled estimate was numerically higher among studies using cut-offs below 1:100, the between-subgroup difference was not statistically significant (χ^2^*_B_* = 2.60, df = 1, *p* = 0.1067). Considerable residual heterogeneity remained within both cut-off categories (I^2^ = 96.2% in each subgroup).

Consistently, MAT cut-off category was not a significant moderator in the univariable meta-regression (β for ≥1:100 versus <1:100 = −0.7639, 95% CI: −1.9192 to 0.3913; χ^2^*_M_* = 1.68, df = 1, *p* = 0.1950). The cut-off category explained only 1.05% of the between-study heterogeneity, and considerable residual heterogeneity persisted (I^2^ = 98.5%; χ^2^*_B_* = 575.52, df = 22, *p* < 0.0001).

### 3.10 Certainty of evidence

The certainty of evidence for the overall pooled seroprevalence and the main subgroup estimates was assessed using an adapted GRADE approach. The certainty of the overall estimate was rated as very low, primarily because of the considerable inconsistency across studies, imprecision of the pooled estimate, and suspected publication bias or small-study effects. The very high heterogeneity (I^2^ = 96.7%), marked variation in study-specific estimates, and funnel plot asymmetry reduced confidence that the pooled prevalence represents a single underlying value applicable across all occupational populations.

The certainty of evidence for the geographic, diagnostic, and occupational subgroup estimates was also rated as very low. The main reasons for downgrading were substantial or considerable residual heterogeneity and imprecision, particularly for estimates based on few studies or presenting wide confidence intervals. The African and European estimates were particularly affected by imprecision, whereas considerable residual heterogeneity was observed in most geographic and diagnostic subgroups. Within the occupational subgroups, the broad composition of some categories and the wide confidence intervals further limited confidence in the estimated prevalence. Complete judgments and their justifications are presented in Supplementary Table S3.

## 4 Discussion

The findings indicate substantial serological evidence of occupational exposure to *Leptospira* spp. across diverse work environments. The pooled global seroprevalence of 18.3% suggests that nearly one in five occupationally exposed workers included in the available literature had detectable anti-*Leptospira* antibodies. However, the considerable heterogeneity observed among studies indicates that this pooled estimate should not be interpreted as a uniform prevalence applicable to all occupational populations. Instead, occupational exposure appears to vary substantially according to geographic, environmental, epidemiological, and study-specific contexts.

The crude aggregated proportion was 21.7%, whereas the random-effects estimate was 18.3%. This difference reflects the distinct weighting structures of the two approaches. The crude proportion treats the dataset as if all participants came from a single population and is therefore driven more strongly by studies with larger sample sizes. In contrast, the random-effects GLMM combines study-specific prevalence estimates on the logit scale and accounts for substantial between-study heterogeneity. Consequently, the model-based estimate is more appropriate for summarizing prevalence across the heterogeneous occupational populations included in this review.

Differences in serological diagnostic methods were formally investigated as a potential source of heterogeneity. Although studies using ELISA combined with MAT showed a numerically higher pooled seroprevalence than those using MAT or ELISA alone, the subgroup difference did not reach statistical significance (*p* = 0.0575). Consistently, diagnostic method was not a significant moderator in meta-regression (*p* = 0.5700; R^2^ = 0%). Thus, the broad diagnostic classification used in this review did not explain a substantial proportion of between-study variability. Nevertheless, differences in antibody targets, cutoff values, antigen or serovar panels, and testing protocols within individual diagnostic categories may still contribute to residual heterogeneity.

A more detailed analysis restricted to MAT studies provided limited evidence that the positivity threshold explained the observed heterogeneity. Studies using cut-offs below 1:100 showed a numerically higher pooled seroprevalence than those using cut-offs of 1:100 or higher (22.6% versus 11.0%), which is consistent with the expectation that less stringent thresholds may classify a greater proportion of participants as seropositive. However, the difference between subgroups was not statistically significant, and cut-off category explained only 1.05% of the between-study heterogeneity in meta-regression. Considerable residual heterogeneity remained within both categories, indicating that the MAT positivity threshold alone did not account for the variability among studies.

Other methodological characteristics of MAT may contribute to the remaining heterogeneity, including the number and composition of serovars included in the panels, the circulation of locally prevalent serovars, laboratory procedures, and criteria for interpreting titres. The serovar panels could not be quantitatively classified into sufficiently homogeneous and mutually comparable groups because their composition was inconsistently reported and varied substantially across studies. Therefore, the influence of serovar panel composition was addressed qualitatively rather than through subgroup analysis or meta-regression.

Individual study estimates varied markedly, ranging from 0 to 100%. Awosanya et al.^16^ reported the highest observed seroprevalence (100%), followed by Abiayi et al. (14) (95.8%), whereas Fernandes et al. (15) reported no seropositive individuals. Bjork et al. (16) and Hashemi et al. (17) reported among the lowest estimates (approximately 1%). The global meta-analysis demonstrated considerable between-study heterogeneity (I^2^ = 96.7%), confirming substantial variability beyond sampling error. Influence diagnostics identified Abiayi et al. (14) as a potentially influential study; however, exclusion of this study reduced the pooled seroprevalence only from 18.3% (95% CI: 11.7–27.4%) to 16.7% (95% CI: 11.1–24.4%), while considerable heterogeneity persisted (I^2^ = 96.1%). These findings indicate that the overall pooled estimate was relatively robust to the exclusion of this influential study and that the observed heterogeneity cannot be attributed to a single study.

Geographic variation emerged as an important moderator in the present study, with pooled seroprevalence estimates varying across continents. However, these regional estimates differed substantially in precision. In particular, the estimate for Europe (2%, 95% CI: 0–9%) was based on only four studies and should therefore be considered imprecise and interpreted with caution. Likewise, the estimate for Africa (27%, 95% CI: 9–58%) was accompanied by a very wide confidence interval and considerable residual heterogeneity (I^2^ = 97.7%), limiting the precision of the pooled estimate. Accordingly, the apparent differences in the magnitude of seroprevalence across continents should not be interpreted as precise rankings of regional disease burden. The formal subgroup test confirmed significant geographic differences (*p* < 0.0001), and continent accounted for approximately 25.7% of between-study heterogeneity in univariable meta-regression. Moreover, after adjustment for publication year, seroprevalence remained significantly lower in the Americas and Europe compared with Asia. These findings suggest that geographic setting captures important differences in endemicity, occupational practices, environmental exposure, socioeconomic conditions, sanitation infrastructure, and potentially methodological characteristics. Nevertheless, substantial residual heterogeneity persisted, and these geographic differences should not be interpreted as evidence of intrinsically higher or lower transmission in any continent.

The considerable heterogeneity observed in the global meta-analysis (I^2^ = 96.7%) indicates substantial variability among the included studies and reinforces that the pooled estimate should not be interpreted as a uniform measure of occupational exposure. Meta-regression provided additional insight into potential sources of this variability. Geographic region accounted for approximately 25.7% of between-study heterogeneity, whereas diagnostic method and occupational category did not explain a measurable proportion of heterogeneity in the models evaluated.

The adapted GRADE assessment indicated very low certainty in the overall and subgroup pooled estimates. This classification does not imply that occupational exposure to *Leptospira* spp. is absent or unimportant. Rather, it indicates limited confidence in the precise magnitude and generalizability of the pooled seroprevalence estimates. The principal factors reducing certainty were the considerable inconsistency across studies, the imprecision of several estimates, and the possibility of small-study effects. Consequently, the overall pooled estimate of 18.3% should be interpreted as an approximate summary of heterogeneous occupational populations and epidemiological settings rather than as a universally applicable prevalence value.

Despite the biological plausibility of different exposure pathways across occupational activities, occupational category was not significantly associated with seroprevalence either in subgroup analysis (*p* = 0.9334) or meta-regression (*p* = 0.9738). This null finding should not be interpreted as evidence that occupational activities are epidemiologically irrelevant. Rather, the broad occupational categories used in the quantitative analysis may have obscured substantial within-category variation in actual exposure. More detailed exposure-related variables were reported in a subset of the included studies, including duration of occupational activity, working hours, use of protective clothing, and specific work tasks or practices. However, these variables were inconsistently defined and reported across studies, precluding their inclusion as separate moderators in the meta-regression. Thus, workers classified within the same broad occupational category may have differed considerably in exposure duration, intensity of contact with potentially contaminated materials, use of personal protective equipment, and performance of specific high-risk tasks. The absence of an association at the occupational-category level may therefore reflect exposure misclassification and insufficient granularity rather than the absence of meaningful differences in occupational risk. Therefore, the absence of a statistically significant difference between the five occupational categories should not be interpreted as evidence of equivalent occupational risk. Rather, the five broad categories may have been too coarse to capture meaningful differences in exposure intensity, duration, use of personal protective equipment, and specific work-related tasks. Future occupational studies should report these exposure characteristics more consistently to enable more granular quantitative analyses.

Several factors may have contributed to this variability. First, the occupational groups differed considerably in the intensity and type of exposure, ranging from agricultural and livestock workers to slaughterhouse, sanitation, market, veterinary, and other occupational groups. These activities involve different degrees of contact with potentially contaminated water, soil, animals, urine, tissues, and waste. Second, substantial differences existed in geographic and environmental conditions, including climate, rainfall, temperature, flooding, sanitation infrastructure, and local epidemiological characteristics. Third, methodological differences among studies, particularly the use of MAT, ELISA, or combinations of serological methods, as well as differences in cutoff values, antigen or serovar panels, sampling strategies, and study designs, may have influenced the classification of seropositive individuals. Finally, differences in sample size and population characteristics may also have contributed to variation in the observed estimates. Therefore, the high heterogeneity likely reflects the combined influence of epidemiological, occupational, environmental, and methodological differences rather than a single underlying source of variability.

This degree of heterogeneity also limits the comparability of prevalence estimates across occupational groups and geographic regions. Although a random-effects model was used to account for between-study variability, the pooled estimate should be interpreted as an overall summary of the available evidence rather than as a representative prevalence applicable to all occupationally exposed workers. The regional estimates should likewise be interpreted cautiously, particularly where the number of contributing studies was small.

Publication year was negatively associated with seroprevalence in univariable meta-regression, suggesting lower estimates in more recently published studies. However, this association was attenuated and no longer statistically significant after adjustment for continent. This finding suggests that the apparent temporal trend may partly reflect differences in the geographic distribution of studies over time rather than an independent decline in occupational exposure. Accordingly, the temporal association should be interpreted cautiously.

Funnel plot asymmetry and Egger’s regression test indicated potential small-study effects. However, the negative direction of the Egger test showed that smaller studies tended to report lower seroprevalence estimates than larger studies. This direction is opposite to the conventional publication-bias pattern in which small studies reporting high estimates are preferentially published. Therefore, the observed asymmetry should not be interpreted as evidence that publication bias inflated the pooled prevalence. It may instead reflect differences in study populations, occupational settings, diagnostic methods, sampling strategies, or the greater instability of prevalence estimates in small samples. Moreover, the considerable between-study heterogeneity may affect the reliability of funnel plot–based methods in this context.

The sensitivity analyses supported the robustness of the overall estimate. After excluding the 11 studies in the lowest quartile of sample size, the pooled seroprevalence decreased modestly from 18.3 to 16.3%, with substantial overlap between the confidence intervals of the primary and sensitivity analyses. In a separate targeted analysis, the simultaneous exclusion of the three studies with extreme observed proportions—Awosanya et al. (18) (10/10), Abiayi et al. (14) (252/263), and Fernandes et al. (44) (0/347)—resulted in a pooled seroprevalence of 17.1%, compared with 18.3% in the primary analysis, while τ^2^ decreased from 2.8823 to 1.5887. Funnel plot asymmetry remained statistically significant after their exclusion. Therefore, although the three studies contributed to between-study variability, neither the smallest studies nor the three studies with extreme proportions were the primary determinants of the pooled estimate. The persistence of heterogeneity and funnel plot asymmetry suggests that these findings reflect broader differences across the included populations, occupational settings, and study characteristics.

In continents with a higher number of studies, such as Asia and Africa, there is also a greater concentration of occupational activities traditionally associated with infection risk, especially agriculture, livestock farming, and work in flooded areas. In Asia, for example, several studies involve rice plantation workers, who are frequently exposed to waterlogged soils and contaminated water. In this review, nine studies were identified in Malaysia alone, as leptospirosis is endemic in the region, with an annual incidence between 1 and 10 cases per 100,000 inhabitants. In addition, population awareness levels directly influence risk: many workers are unaware of transmission routes and do not consistently or properly use of protective clothing, increasing occupational exposure (18–21).

The growth of ecotourism and the expansion of agriculture into natural areas also play a relevant role in occupational leptospirosis dynamics, as they increase the interface between humans, domestic animals, and wildlife. Park workers, reserve staff, adventure activity workers, as well as rural producers and workers in intensive farming systems, increasingly operate in environments with higher circulation of reservoirs and greater soil and water contamination, especially in tropical and flood-prone regions. Deforestation, habitat fragmentation, and inadequate water management favor environmental persistence of *Leptospira*, intensifying occupational risk and modifying disease epidemiological patterns, requiring integrated surveillance and territorial planning under a One Health perspective (22, 23).

A wide variation in individual seroprevalence estimates was observed across studies, ranging from 0 to 100%. Considerable heterogeneity was also observed within geographic subgroups, although its magnitude varied across continents. Heterogeneity was highest in Africa (I^2^ = 97.7%), followed by Oceania (I^2^ = 95.7%), the Americas (I^2^ = 94.6%), and Asia (I^2^ = 93.0%), while a lower, although still substantial, level of heterogeneity was observed in Europe (I^2^ = 72.8%). These findings indicate that substantial variability remained even after stratification by geographic region. Such variations may be attributed to differences in environmental, climatic, and socioeconomic contexts, as well as types of occupational activity performed. Workers involved in agriculture, livestock farming, sanitation, waste management, and slaughterhouses tend to present higher exposure risk, especially in tropical and subtropical regions, where high temperatures and humidity favor bacterial survival in the environment (24, 25),.

In this context, the One Health approach is essential for addressing leptospirosis by integrating human, animal, and environmental factors. Preventive measures such as personal protective equipment use, health education, and animal reservoir control are fundamental to reducing disease burden (6). The findings of this review reinforce the need for targeted public policies for at-risk groups and methodological standardization in diagnostic approaches to produce more accurate global estimates. From an occupational health perspective, preventive strategies should include regular training on *Leptospira* transmission and exposure risks, appropriate use of personal protective equipment (PPE), access to safe water and sanitation facilities, proper wound protection, and measures to reduce contact with potentially contaminated water, soil, animal tissues, urine, and waste. Periodic health surveillance and targeted risk communication should also be prioritized for workers in high-risk occupations, particularly those involved in agriculture, livestock handling, slaughterhouses, sanitation, and waste management.

From an occupational health policy perspective, the findings support the need for strengthened surveillance and preventive measures, but the very low certainty of the pooled estimates limits their use as precise quantitative benchmarks for resource allocation or comparisons between regions and occupational groups. Policy decisions should therefore consider local epidemiological data, occupational practices, environmental conditions, diagnostic strategies, and surveillance capacity. The pooled and subgroup estimates are more appropriately interpreted as evidence of substantial and geographically variable occupational exposure than as precise estimates of burden applicable to every setting.

### 4.1 Limitations

This study has some limitations. The very high between-study heterogeneity (I^2^ = 96.7%) limits the comparability and generalizability of the pooled estimates. Differences in occupational populations, geographic and environmental conditions, sampling strategies, and serological diagnostic methods may have contributed to this variability. In addition, the number of studies varied considerably among regions, and some regional estimates were characterized by limited precision. In particular, the European estimate was based on only four studies and had a wide confidence interval (2%, 95% CI: 0–9%), while the African estimate also showed substantial uncertainty (27%, 95% CI: 9–58%) and very high residual heterogeneity. Therefore, regional estimates should be interpreted cautiously and should not be considered precise rankings of disease burden or representative of all occupational groups and geographic settings.

Additionally, the predefined 15-year publication window excluded older studies that might have provided additional information on occupational seroprevalence. Although this restriction was intended to prioritize more contemporary epidemiological evidence, it may have reduced the comprehensiveness of the available evidence and should be considered when interpreting the findings.

Furthermore, the adapted GRADE assessment indicated very low certainty for the overall and subgroup estimates. This classification was driven mainly by considerable inconsistency, imprecision, and potential small-study effects. Although the assessment provides a transparent evaluation of confidence in the estimates, GRADE was originally developed primarily for evaluating intervention effects, and its application to prevalence estimates requires adapted judgments. Therefore, the certainty ratings should be interpreted as structured assessments of confidence in the estimated prevalence rather than as evaluations of intervention effectiveness.

Furthermore, the MAT cut-off was not identifiable in four of the 28 MAT studies, and the broad categorization of titres as <1:100 or ≥1:100 may not capture all differences in diagnostic interpretation. The composition of the serovar panels could not be quantitatively assessed because of heterogeneous and incomplete reporting. These limitations may have reduced the ability of the subgroup analysis and meta-regression to identify diagnostic sources of heterogeneity.

## 5 Conclusion

This systematic review and meta-analysis indicates substantial serological evidence of exposure to *Leptospira* spp. among occupationally exposed workers worldwide, with a pooled seroprevalence of 18.3% (95% CI: 11.7–27.4%). Geographic region emerged as an important source of between-study variability, whereas broad diagnostic and occupational categories did not significantly explain heterogeneity. Although publication year was negatively associated with seroprevalence in univariable meta-regression, this association was attenuated after adjustment for geographic region, indicating that the apparent temporal trend may partly reflect geographic differences among studies. Considerable residual heterogeneity remained, and the certainty of the pooled and subgroup estimates was rated as very low, primarily because of inconsistency, imprecision, and potential small-study effects. Therefore, the pooled estimates should be interpreted cautiously and should not be considered universally applicable measures of occupational burden. These findings reinforce the need for standardized serological surveillance, more detailed characterization of occupational exposures, and targeted preventive strategies integrating human, animal, and environmental health within a One Health framework.

## Data Availability

The original contributions presented in the study are included in the article/Supplementary material, further inquiries can be directed to the corresponding author/s.

## Supplementary material

SUPPLEMENTARY FIGURE S1 Forest plot of Leptospira seroprevalence among occupationally exposed populations, stratified by microscopic agglutination test (MAT) cutoff titers (<1:100 and ≥1:100). Diamonds represent pooled seroprevalence estimates from random-effects models, with 95% confidence intervals.

## References

[1] Raji AO Lawal HA Suleiman ZA Olayiwola AE Muiz W Tinuke OP . Integrative one health strategies for the surveillance, control, and prevention of vector-borne and zoonotic diseases: bridging human, animal, and environmental health in a changing world. J Glob Health Sci. (2026) 8:e2. doi: 10.35500/jghs.2026.8.e2

[2] One Health High-Level Expert Panel (OHHLEP) Adisasmito WB Almuhairi S Behravesh CB Bilivogui P Bukachi SA Casas N . One health: a new definition for a sustainable and healthy future. PLoS Pathog. (2022) 18:e1010537. doi: 10.1371/journal.ppat.1010537, 35737670 PMC9223325

[3] Hartskeerl RA Collares-Pereira M Ellis WA. Emergence, control and re-emerging leptospirosis: dynamics of infection in the changing world. Clin Microbiol Infect. (2011) 17:494–501. doi: 10.1111/j.1469-0691.2011.03474.x, 21414083

[4] Su H Xu K Wang W Liu Y Ping G Bo D. Unveiling *Leptospira* prevalence and exposure in sanitation workers, a cross-sectional study in Ningbo City, China. Front Public Health. (2025) 13:1627155. doi: 10.3389/fpubh.2025.1627155, 40672915 PMC12263941

[5] Silva GM Oliveira JMB Silva Neto AL Assis NA Mathias LA Brandespim DF . Pesquisa de anticorpos anti-Leptospira spp. em grupos ocupacionais no estado de Pernambuco. Rev Inst Adolfo Lutz. (2015) 73:252–8. doi: 10.18241/0073-98552014731612

[6] Mendes J Aymée L Lilenbaum W. A scoping review of antimicrobial therapy in Leptospira infections in domestic animals. Animals. (2025) 15:3045. doi: 10.3390/ani15203045, 41153972 PMC12561877

[7] Giraud-Gatineau A Dagbo K Pětrošová H Werts C Veyrier FJ Picardeau M. Shaping the future of Leptospira serotyping. J Med Microbiol. (2025) 74:002059. doi: 10.1099/jmm.0.002059, 40938636 PMC12451757

[8] Pan American Health Organization Leptospirosis. Washington, DC, United States: Pan American Health Organization (PAHO) (2026).

[9] Torgerson PR Hagan JE Costa F Calcagno J Kane M Martinez-Silveira MS . Global burden of leptospirosis: estimated in terms of disability adjusted life years. PLoS Negl Trop Dis. (2015) 9:e0004122. doi: 10.1371/journal.pntd.0004122, 26431366 PMC4591975

[10] Pinto GV Senthilkumar K Rai P Kabekkodu SP Karunasagar I Kumar BK. Current methods for the diagnosis of leptospirosis: issues and challenges. J Microbiol Methods. (2022) 195:106438. doi: 10.1016/j.mimet.2022.106438, 35248601

[11] Arboleda M Mejía-Torres M Posada M Restrepo N Ríos-Tapias P Rivera-Pedroza LA . Molecular diagnosis as an alternative for public health surveillance of leptospirosis in Colombia. Microorganisms. (2023) 11:2759. doi: 10.3390/microorganisms11112759, 38004770 PMC10673046

[12] Valente M Bramugy J Keddie SH Hopkins H Bassat Q Baerenbold O . Diagnosis of human leptospirosis: systematic review and meta-analysis of the diagnostic accuracy of the Leptospira microscopic agglutination test, PCR targeting Lfb1, and IgM ELISA to *Leptospira fainei* serovar Hurstbridge. BMC Infect Dis. (2024) 24:168. doi: 10.1186/s12879-023-08935-0, 38326762 PMC10848445

[13] Page MJ McKenzie JE Bossuyt PM Boutron I Hoffmann TC Mulrow CD . The PRISMA 2020 statement: an updated guideline for reporting systematic reviews. BMJ. (2020) 372:n71. doi: 10.1136/bmj.n71, 33782057 PMC8005924

[14] Abiayi EA Inabo HI Jatau ED Makinde AA Sar TT Ugbe DA . Knowledge, attitudes, risk factors and practices (KARP) that favor *Leptospira* infection among abattoir workers in north Central Nigeria. Asian J Epidemiol. (2015) 8:104–13. doi: 10.3923/aje.2015.104.113

[15] Hashemi SA Arzamani K Abdollahpour G Beheshti N Alavinia M Azimian A . Seroprevalence of *Leptospira* infection in occupational risk groups in North Khorasan province, Iran. Heliyon. (2021) 7:e05983. doi: 10.1016/j.heliyon.2021.e05983, 33506135 PMC7814158

[16] Binti Daud A Mohd Fuzi NMH Wan Mohammad WMZ Amran F Ismail N Arshad MM . Leptospirosis and workplace environmental risk factors among cattle farmers in northeastern Malaysia. Int J Occup Environ Med. (2018) 9:88–96. doi: 10.15171/ijoem.2018.1164 PMC6466985 29667646

[17] Jamora RM Villanueva SYAM Gloriani NG. Seroprevalence of *Leptospira* and description of work-related practices and characteristics among sewer workers servicing selected cities in the west zone of metro Manila, Philippines. Philipp J Sci. (2022) 151:215–26. doi: 10.56899/151.01.16

[18] Awosanya EJ Nguku P Oyemakinde A Omobowale O. Factors associated with probable cluster of leptospirosis among kennel workers in Abuja, Nigeria. Pan Afr Med J. (2013) 16:144. doi: 10.11604/pamj.2013.16.144.3529, 24711882 PMC3972904

[19] Goh SH Ismail R Lau SF Megat Abdul Rani PA Mohd Mohidin TB Daud F . Risk factors and prediction of leptospiral seropositivity among dogs and dog handlers in Malaysia. Int J Environ Res Public Health. (2019) 16., 31035316 10.3390/ijerph16091499 PMC6540030

[20] El Azhari M Picardeau M Cherkaoui I Sadat MA Moumni H Marhoum El Filali K . Seroprevalence of leptospirosis among high-risk individuals in Morocco. Interdiscip Perspect Infect Dis. (2020) 2020:1–8. doi: 10.1155/2020/5236045, 32565788 PMC7256772

[21] Bjork A Stoddard RA Anderson AD de Perio MA Niemeier RT Self JS . Zoonoses in the workplace: a seroprevalence study of *Coxiella*, *Brucella*, and *Leptospira* among marine mammal rescue and rehabilitation workers in California. Public Health Chall. (2024) 3:e132. doi: 10.1002/puh2.132, 40162119 PMC11951298

[22] Mohd Ridzuan J Aziah BD Zahiruddin WM. Work environment-related risk factors for leptospirosis among plantation Workers in Tropical Countries: evidence from Malaysia. Int J Occup Environ Med. (2016) 7:156–63. doi: 10.15171/ijoem.2016.699, 27393322 PMC6818082

[23] Garba B Bahaman AR Bejo SK Zakaria Z Mutalib AR Bande F. Major epidemiological factors associated with leptospirosis in Malaysia. Acta Trop. (2018) 178:242–7. doi: 10.1016/j.actatropica.2017.12.010, 29217379

[24] Bandara M Ananda M Wickramage K Berger E Agampodi S. Globalization of leptospirosis through travel and migration. Glob Health. (2014) 10:61. doi: 10.1186/s12992-014-0061-0, 25112368 PMC4131158

[25] Wang W Gao Y Ji J Huang Z Xiong B Xiang S. Trends and advances in *Leptospira*, a bibliometric analysis. Front Microbiol. (2024) 15:1514738. doi: 10.3389/fmicb.2024.1514738, 39845041 PMC11750782

[26] Majd NS Darian EK Khaki P Bidhendi SM Yahaghi E Mirnejad R. Epidemiological patterns of *Leptospira* spp. among slaughterhouse workers in Zanjan–Iran. Asian Pac J Trop Dis. (2012) 2:S550–2. doi: 10.1016/S2222-1808(12)60218-7

[27] Shafei MN Sulong MR Yaacob NA Hassan H Wan Mohamad WMZ Daud A . Seroprevalence of leptospirosis among town service workers in northeastern state of Malaysia. Int J Collab Res Intern Med Public Health. (2012) 4:395–403.

[28] Alavi SM Khoshkho MM. Seroprevalence study of leptospirosis among rice farmers in Khuzestan Province, south West Iran, 2012. Jundishapur J Microbiol. (2014) 7:e11536. doi: 10.5812/jjm.11536, 25368799 PMC4216579

[29] Alvarado-Esquivel C Hernandez-Tinoco J Sanchez-Anguiano LF Ramos-Nevarez A Cerrillo-Soto SM Guido-Arreola CA. *Leptospira* exposure and waste pickers: a case-control seroprevalence study in Durango, Mexico. J Clin Med Res. (2015) 7:637–40. doi: 10.14740/jocmr2217w, 26124911 PMC4471752

[30] López F Samudio M de Assis DM Cabello A. Seroprevalencia de leptospirosis y factores asociados en trabajadores del servicio de aseo urbano de la Municipalidad de Asunción, Paraguay. Rev Chilena Infectol. (2015) 32:628–33. doi: 10.4067/S0716-10182015000700003, 26928498

[31] Samsudin S Masri SN Tengku Jamaluddin TZM Saudi SNS Md Ariffin UK Amran F . Seroprevalence of leptospiral antibodies among healthy municipal service workers in Selangor. Adv Public Health. (2015) 2015:208145. doi: 10.1155/2015/208145

[32] Sanhueza JM Heuer C Wilson PR Benschop J Collins-Emerson JM. Prevalence and risk factors for Leptospira exposure in New Zealand veterinarians. Epidemiol Infect. (2015) 143:2116–25. doi: 10.1017/S0950268815000515, 25804406 PMC9506997

[33] Alvarado-Esquivel C Hernandez-Tinoco J Sanchez-Anguiano LF Ramos-Nevarez A Cerrillo-Soto SM Saenz-Soto L . High seroprevalence of Leptospira exposure in meat workers in northern Mexico: a case-control study. J Clin Med Res. (2016) 8:231–6. doi: 10.14740/jocmr2463w, 26858797 PMC4737035

[34] Dreyfus A Dyal JW Pearson R Kankya C Kajura C Alinaitwe L . Leptospira seroprevalence and risk factors in health Centre patients in Hoima District, Western Uganda. PLoS Negl Trop Dis. (2016) 10:e0004858. doi: 10.1371/journal.pntd.0004858, 27487398 PMC4972303

[35] Esmaeili S Naddaf SR Pourhossein B Hashemi Shahraki A Bagheri Amiri F Gouya MM . Seroprevalence of brucellosis, leptospirosis, and Q fever among butchers and slaughterhouse workers in south-eastern Iran. PLoS One. (2016) 11:e0144953. doi: 10.1371/journal.pone.0144953, 26731333 PMC4701462

[36] Guzmán-Barragán BL Peña-Perdomo SM Flórez-Rojas RF Amado-Gómez DF Rodríguez-Ruíz EM Verjan-García N. Prevalencia de anticuerpos anti-Leptospira spp. en personas con exposición laboral en el departamento del Tolima. Rev Fac Nac Salud Publica. (2016) 34:156–66. doi: 10.17533/udea.rfnsp.v34n2a04

[37] Parveen SMA Suganyaa B Sathya MS Margreat AAP Sivasankari K Shanmughapriya S . Leptospirosis seroprevalence among blue metal mine workers of Tamil Nadu, India. Am J Trop Med Hyg. (2016) 95:38–42. doi: 10.4269/ajtmh.16-0095, 27044567 PMC4944705

[38] Cook EAJ de Glanville WA Thomas LF Kariuki S Bronsvoort BMDC Fèvre EM. Risk factors for leptospirosis seropositivity in slaughterhouse workers in western Kenya. Occup Environ Med. (2017) 74:357–65. doi: 10.1136/oemed-2016-103895, 27913579 PMC5520261

[39] Pittavino M Dreyfus A Heuer C Benschop J Wilson P Collins-Emerson J . Comparison between generalized linear modelling and additive Bayesian network; identification of factors associated with the incidence of antibodies against *Leptospira interrogans* sv Pomona in meat workers in New Zealand. Acta Trop. (2017) 173:191–9. doi: 10.1016/j.actatropica.2017.04.034, 28487178

[40] Mirambo MM Mgode GF Malima ZO John M Mngumi EB Mhamphi GG . Seropositivity of *Brucella* spp. and *Leptospira* spp. antibodies among abattoir workers and meat vendors in the city of Mwanza, Tanzania: a call for one health approach control strategies. PLoS Negl Trop Dis. (2018) 12:e0006600. doi: 10.1371/journal.pntd.0006600, 29939991 PMC6034905

[41] Samsudin S Sakinah SNS Malina O Norliza BA Noh MA Fairuz A . Seroprevalence of leptospiral antibodies among market workers and food handlers in the central state of Malaysia. Trop Med Int Health. (2018) 23:327–33. doi: 10.1111/tmi.13033, 29356240

[42] Rahman MHAA Hairon SM Hamat RA Jamaluddin TZMT Idris N Osman M . Seroprevalence and distribution of leptospirosis serovars among wet market workers in northeastern, Malaysia: a cross sectional study. BMC Infect Dis. (2018) 18:569. doi: 10.1186/s12879-018-3470-5, 30428852 PMC6236877

[43] Ataya GE Pedraza-Bernal AM Vargas DC Romero LY Jaimes-Bernal CP Merchán-Castellanos NA. Anti-*Leptospira* spp. antibodies in meat dealers in Tunja, Boyacá, Colombia. J Bras Patol Med Lab. (2019) 55:122–35. doi: 10.5935/1676-2444.20190013

[44] Fernandes M Vieira ML Carreira T Teodósio R. Sanitation workers from Portugal: is there evidence of *Leptospira* spp.? J Infect Public Health. (2019) 12:738–40. doi: 10.1016/j.jiph.2019.02.001, 30777720

[45] Mgode GF Japhary MM Mhamphi GG Kiwelu I Athaide I Machang'u RS. Leptospirosis in sugarcane plantation and fishing communities in Kagera northwestern Tanzania. PLoS Negl Trop Dis. (2019) 13:e0007225. doi: 10.1371/journal.pntd.0007225, 31150391 PMC6544212

[46] Taus K Schmoll F El-Khatib Z Auer H Holzmann H Aberle S . Occupational swine exposure and hepatitis E virus, *Leptospira*, *Ascaris suum* seropositivity and MRSA colonization in Austrian veterinarians, 2017–2018—a cross-sectional study. Zoonoses Public Health. (2019) 66:842–51. doi: 10.1111/zph.12633, 31419070 PMC6851874

[47] Yih WK Kulldorff M Friedman DJ Leibler JH Amador JJ López-Pilarte D . Investigating possible infectious causes of chronic kidney disease of unknown etiology in a Nicaraguan mining community. Am J Trop Med Hyg. (2019) 101:676–83. doi: 10.4269/ajtmh.18-0856, 31309920 PMC6726956

[48] Goh SH Khor KH Ismail R Megat Abdul Rani PA Mohd Mohidin TB Bahaman AR . Detection and distribution of anti-leptospiral antibody among dogs and their handlers. Trop Biomed. (2020) 37:1074–82. doi: 10.47665/tb.37.4.1074, 33612759

[49] Gómez-Martín M Lozano C Luque R Luque-Romero L Rodríguez-Benjumeda L Aznar-Martín J. Red swamp crayfish collecting: a risk activity for leptospirosis. Clin Microbiol Infect. (2020) 26:1103–4. doi: 10.1016/j.cmi.2020.03.009, 32224201

[50] Jeffree MS Mori D Yusof NA Atil AB Awang Lukman K Othman R . High incidence of asymptomatic leptospirosis among urban sanitation workers from Kota Kinabalu, Sabah, Malaysian Borneo. Sci Rep. (2020) 10:19442. doi: 10.1038/s41598-020-76595-0, 33173153 PMC7655852

[51] Murcia CA Astudillo M Romero MH. Prevalence of leptospirosis in vaccinated working dogs and humans with occupational risk. Biomedica. (2020) 40:62–75. doi: 10.7705/biomedica.5009, 32463609 PMC7449099

[52] Ntabanganyimana E Giraneza R Dusabejambo V Bizimana A Hamond C Iyamuremye A . Sero-prevalence of anti-Leptospira antibodies and associated risk factors in rural Rwanda: a cross-sectional study. PLoS Negl Trop Dis. (2021) 15:e0009708. doi: 10.1371/journal.pntd.0009708, 34874936 PMC8683035

[53] Meny P Iglesias T Menéndez C Quintero J Ríos C Ashfield N . Seroprevalence of anti-Leptospira antibodies in equines and associated workers—isolation of *Leptospira interrogans* serogroup Canicola from equine urine. Zoonoses Public Health. (2022) 69:526–36. doi: 10.1111/zph.12942, 35347868

[54] Zida S Ouédraogo HG Compaoré TR Sagna T Soubeiga ST Banhoro BY . Leptospirosis seroprevalence and risk factors among slaughterhouse workers in Burkina Faso. IJID Reg. (2023) 9:125–30. doi: 10.1016/j.ijregi.2023.10.008, 38074946 PMC10701108

[55] Klier CM Princk C Richter MH Luge E Mayer-Scholl A Mylius M . Anti-Leptospira Seroprevalence and associated risk factors among forestry Workers in Lower Saxony, north-West Germany. Microorganisms. (2024) 12:1262. doi: 10.3390/microorganisms12071262, 39065028 PMC11279034

[56] Onafruo D Dreyfus A Erume J Kankya C Jubara A Kokas I . Leptospira seroprevalence and associated risk factors among slaughterhouse workers in Western Bahr El Ghazal state, South Sudan. PLoS Negl Trop Dis. (2024) 18:e0012700. doi: 10.1371/journal.pntd.0012700, 39661611 PMC11633975

[57] Mevada Y Vinod KK Balamurgan V Chavhan S Kumar J Palkhade R. Seroprevalence and risk factors associated with leptospirosis in high-risk occupational groups in the state of Gujarat as determined by IgM ELISA and MAT test: a cross-sectional study. Indian J Occup Environ Med. (2024) 28:106–14. doi: 10.4103/ijoem.ijoem_83_23, 39114107 PMC11302540

