# Supplementary material for: Global seroprevalence of Leptospira spp. infection in workers with occupational risk: a systematic review with meta-analysis
Source: Front Public Health. 2026 Oct 21;14:1931298. doi: 10.3389/fpubh.2026.1931298 (PMC13731361; doi:10.3389/fpubh.2026.1931298)
Supplement: Supplementary file 1 [file Image_1.pdf]

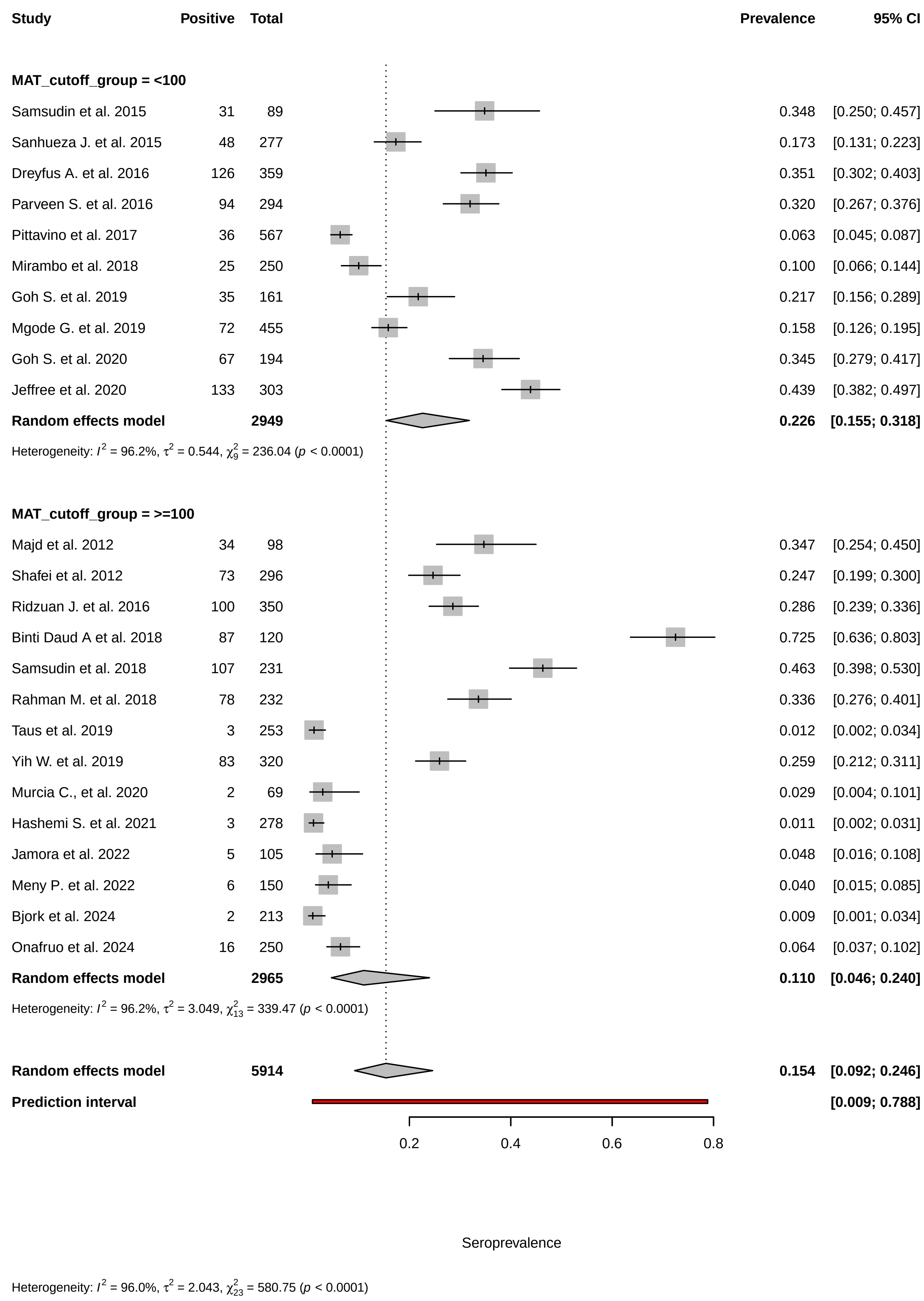
Study Positive Total Prevalence 95% CI
MAT_cutoff_group = <100
Samsudin et al. 2015 31 89 0.348 [0.250; 0.457]
Sanhueza J. et al. 2015 48 277 0.173 [0.131; 0.223]
Dreyfus A. et al. 2016 126 359 0.351 [0.302; 0.403]
Parveen S. et al. 2016 94 294 0.320 [0.267; 0.376]
Pittavino et al. 2017 36 567 0.063 [0.045; 0.087]
Mirambo et al. 2018 25 250 0.100 [0.066; 0.144]
Goh S. et al. 2019 35 161 0.217 [0.156; 0.289]
Mgode G. et al. 2019 72 455 0.158 [0.126; 0.195]
Goh S. et al. 2020 67 194 0.345 [0.279; 0.417]
Jeffree et al. 2020 133 303 0.439 [0.382; 0.497]
Random effects model 2949 0.226 [0.155; 0.318]
Heterogeneity: $I^2 = 96.2\%$, $\tau^2 = 0.544$, $\chi^2_9 = 236.04$ ($p < 0.0001$)
MAT_cutoff_group = >=100
Majd et al. 2012 34 98 0.347 [0.254; 0.450]
Shafei et al. 2012 73 296 0.247 [0.199; 0.300]
Ridzuan J. et al. 2016 100 350 0.286 [0.239; 0.336]
Binti Daud A et al. 2018 87 120 0.725 [0.636; 0.803]
Samsudin et al. 2018 107 231 0.463 [0.398; 0.530]
Rahman M. et al. 2018 78 232 0.336 [0.276; 0.401]
Taus et al. 2019 3 253 0.012 [0.002; 0.034]
Yih W. et al. 2019 83 320 0.259 [0.212; 0.311]
Murcia C., et al. 2020 2 69 0.029 [0.004; 0.101]
Hashemi S. et al. 2021 3 278 0.011 [0.002; 0.031]
Jamora et al. 2022 5 105 0.048 [0.016; 0.108]
Meny P. et al. 2022 6 150 0.040 [0.015; 0.085]
Bjork et al. 2024 2 213 0.009 [0.001; 0.034]
Onafruo et al. 2024 16 250 0.064 [0.037; 0.102]
Random effects model 2965 0.110 [0.046; 0.240]
Heterogeneity: $I^2 = 96.2\%$, $\tau^2 = 3.049$, $\chi^2_{13} = 339.47$ ($p < 0.0001$)
Random effects model 5914 0.154 [0.092; 0.246]
Prediction interval [0.009; 0.788]
0.2
0.4
0.6
0.8
Seroprevalence
Heterogeneity: $I^2 = 96.0\%$, $\tau^2 = 2.043$, $\chi^2_{23} = 580.75$ ($p < 0.0001$)
