# Supplementary material for: Global seroprevalence of Leptospira spp. infection in workers with occupational risk: a systematic review with meta-analysis
Source: Front Public Health. 2026 Oct 21;14:1931298. doi: 10.3389/fpubh.2026.1931298 (PMC13731361; doi:10.3389/fpubh.2026.1931298)
Supplement: Supplementary file 2 [file Table_1.pdf]

Supplementary Table 1. Complete search strategies used for each electronic database included in the systematic review.

Database	Complete search strategy
PubMed	("Leptospirosis"[Mesh] OR leptospirosis) AND ("Occupational Diseases"[Mesh] OR occupational disease* OR occupation* OR worker*) AND (prevalence OR serodiagnosis)
Scopus	TITLE-ABS-KEY(leptospirosis AND occupational disease* AND (prevalence OR serodiagnosis))
Web of Science	TS=(leptospirosis AND occupational disease* AND (prevalence OR serodiagnosis))
MEDLINE	(Leptospirosis[MeSH Terms] OR leptospirosis) AND (Occupational Diseases[MeSH Terms] OR occupational disease* OR occupation* OR worker*) AND (prevalence OR serodiagnosis)
LILACS	(mh:(Leptospirosis) OR leptospirosis) AND (mh:("Occupational Diseases") OR "occupational disease*" OR occupation* OR worker*) AND (prevalence OR serodiagnosis)
ScienceDirect	leptospirosis AND ("occupational disease" OR occupation* OR worker*) AND (prevalence OR serodiagnosis)
