# Supplementary material for: Global seroprevalence of Leptospira spp. infection in workers with occupational risk: a systematic review with meta-analysis
Source: Front Public Health. 2026 Oct 21;14:1931298. doi: 10.3389/fpubh.2026.1931298 (PMC13731361; doi:10.3389/fpubh.2026.1931298)
Supplement: Supplementary file 3 [file Table_2.pdf]

Supplementary Table S2. Methodological quality assessment of the included studies using the Joanna Briggs Institute (JBI) Critical Appraisal Checklist for Studies Reporting Prevalence Data.

**Study**	**q1**	**q2**	**q3**	**q4**	**q5**	**q6**	**q7**	**q8**	**q9**	**Score**	**Quality**
Shafei et al. 2012	Yes	Yes	Yes	Yes	Yes	Yes	Yes	Yes	Yes	9	High
Majd et al. 2012	Yes	Yes	No	Yes	Yes	Yes	Yes	Yes	Yes	8	High
Awosanya et al. 2013	Yes	Yes	No	Yes	Yes	Yes	Yes	Yes	No	7	High
Alavi et al. 2014	Yes	Yes	Yes	Yes	Yes	Yes	Yes	Yes	Yes	9	High
Abiayi et al. 2015	Yes	Yes	No	Yes	Yes	Yes	Yes	Yes	Yes	8	High
Alvarado-Esquivel et al. 2015	Yes	Yes	No	Yes	Yes	Yes	Yes	Yes	Yes	8	High
Lopez et al. 2015	Yes	Yes	Yes	Yes	Yes	Yes	Yes	Yes	Yes	9	High
Samsudin et al. 2015	Yes	Yes	No	Yes	Yes	Yes	Yes	Yes	Yes	8	High
Sanhueza et al. 2015	Yes	Yes	No	Yes	Yes	Yes	Yes	Yes	Yes	8	High

Alvarado-Esquivel et al. 2016	Yes	Yes	Yes	Yes	Yes	Yes	Yes	Yes	Yes	9	High
Gusmán-Barragán et al. 2016	Yes	Yes	No	Yes	Yes	Yes	Yes	Yes	Yes	8	High
Cook et al. 2017	Yes	Yes	Yes	Yes	Yes	Yes	Yes	Yes	Yes	9	High
Dreyfus et al. 2016	Yes	Yes	Yes	Yes	Yes	Yes	Yes	Yes	No	8	High
Esmaeili et al. 2016	Yes	Yes	No	Yes	Yes	Yes	Yes	Yes	Yes	8	High
Parveen et al. 2016	Yes	Yes	Yes	Yes	Yes	Yes	Yes	Yes	Yes	9	High
Ridzuan et al. 2016	Yes	Yes	No	Yes	Yes	Yes	Yes	Yes	Yes	8	High
Pittavino et al. 2017	Yes	Yes	Yes	Yes	Yes	Yes	Yes	Yes	No	8	High
Daud et al. 2018	Yes	Yes	Yes	Yes	Yes	Yes	Yes	Yes	Yes	9	High
Mirambo et al. 2018	Yes	Yes	Yes	Yes	Yes	Yes	Yes	Yes	Yes	9	High
Rahman et al. 2018	Yes	Yes	Yes	Yes	Yes	Yes	Yes	Yes	Yes	9	High

Samsudin et al. 2018	Yes	Yes	Yes	Yes	Yes	Yes	Yes	Yes	Yes	9	High
Ataya et al. 2019	Yes	Yes	Yes	Yes	Yes	Yes	Yes	Yes	Yes	9	High
Fernandes et al. 2019	Yes	Yes	Yes	Yes	Yes	Yes	Yes	Yes	Yes	9	High
Goh et al. 2019	Yes	Yes	No	Yes	Yes	Yes	Yes	Yes	Yes	8	High
Mgode et al. 2019	Yes	Yes	Yes	Yes	Yes	Yes	Yes	Yes	Yes	9	High
Taus et al. 2019	Yes	Yes	Yes	Yes	Yes	Yes	Yes	Yes	Yes	9	High
Yih et al. 2019	Yes	No	Yes	Yes	Yes	Yes	Yes	Yes	Yes	8	High
Azhari et al. 2020	Yes	Yes	Yes	Yes	Yes	Yes	Yes	Yes	Yes	9	High
Gomez-Martin et al. 2020	Yes	Yes	Yes	Yes	Yes	Yes	Yes	Yes	Yes	9	High
Goh et al. 2020	Yes	Yes	Yes	Yes	Yes	Yes	Yes	Yes	Yes	9	High
Jeffree et al. 2020	Yes	Yes	No	Yes	Yes	Yes	Yes	Yes	Yes	8	High

Murcia et al. 2020	Yes	Yes	No	Yes	Yes	Yes	Yes	Yes	Yes	8	High
Hashemi et al. 2021	Yes	Yes	No	Yes	Yes	Yes	Yes	Yes	Yes	8	High
Ntabanganyimana et al. 2021	Yes	Yes	No	Yes	Yes	Yes	Yes	Yes	Yes	8	High
Jamora et al. 2022	Yes	Yes	No	Yes	Yes	Yes	Yes	Yes	Yes	8	High
Meny et al. 2022	Yes	No	Yes	Yes	Yes	Yes	Yes	Yes	Yes	8	High
Zida et al. 2023	Yes	Yes	No	Yes	Yes	Yes	Yes	Yes	Yes	8	High
Bjork et al. 2024	Yes	No	No	Yes	Yes	Yes	Yes	Yes	Yes	8	High
Klier et al. 2024	Yes	Yes	No	Yes	Yes	Yes	Yes	Yes	Yes	8	High
Mevada et al. 2024	Yes	Yes	Yes	Yes	Yes	Yes	Yes	Yes	Yes	9	High
Onafruo et al. 2024	Yes	Yes	Yes	Yes	Yes	Yes	Yes	Yes	Yes	9	High
Su et al. 2025	Yes	Yes	No	Yes	Yes	Yes	Yes	Yes	Yes	8	High
