# Supplementary material for: Global seroprevalence of Leptospira spp. infection in workers with occupational risk: a systematic review with meta-analysis
Source: Front Public Health. 2026 Oct 21;14:1931298. doi: 10.3389/fpubh.2026.1931298 (PMC13731361; doi:10.3389/fpubh.2026.1931298)
Supplement: Supplementary file 4 [file Table_3.pdf]

Supplementary Table S3. Certainty of evidence for the overall and subgroup pooled seroprevalence estimates using an adapted GRADE approach.

Estimate	Pooled prevalence (95% CI)	Risk of bias	Inconsistency	Indirectness	Imprecision	Publication bias	Certainty
Overall	18.3% (11.7–27.4%)	Not serious[1]	Very serious[2]	Not serious[3]	Serious[4]	Serious concern regarding small-study effects[5]	⊕◯◯◯ Very low
Asia	33% (25–42%)	Not serious[1]	Very serious[6]	Not serious[3]	Not serious	Not assessed[7]	⊕◯◯◯ Very low
Africa	27% (9–58%)	Not serious[1]	Very serious[8]	Not serious[3]	Very serious[9]	Not assessed[7]	⊕◯◯◯ Very low
Oceania	11% (5–21%)	Not serious[1]	Very serious[10]	Not serious[3]	Serious[11]	Not assessed[7]	⊕◯◯◯ Very low
Americas	9% (4–20%)	Not serious[1]	Very serious[12]	Not serious[3]	Serious[11]	Not assessed[7]	⊕◯◯◯ Very low
Europe	2% (0–9%)	Not serious[1]	Serious[13]	Not serious[3]	Very serious[14]	Not assessed[7]	⊕◯◯◯ Very low
MAT	18% (9–32%)	Not serious[1]	Very serious[15]	Not serious[3]	Serious[11]	Not assessed[7]	⊕◯◯◯ Very low
ELISA	14% (8–24%)	Not serious[1]	Very serious[15]	Not serious[3]	Serious[11]	Not assessed[7]	⊕◯◯◯ Very low

ELISA combined with MAT	29% (20–40%)	Not serious[1]	Very serious[15]	Not serious[3]	Serious[16]	Not assessed[7]	⊕◯◯◯ Very low
Animal-related occupations	20.9% (10.7–36.9%)	Not serious[1]	Serious[17]	Not serious[3]	Serious[11]	Not assessed[7]	⊕◯◯◯ Very low
Sanitation/waste workers	11.4% (2.9–35.7%)	Not serious[1]	Serious[17]	Not serious[3]	Very serious[18]	Not assessed[7]	⊕◯◯◯ Very low
Agriculture/fishing workers	17.8% (11.3–26.8%)	Not serious[1]	Serious[17]	Not serious[3]	Serious[11]	Not assessed[7]	⊕◯◯◯ Very low
Mixed/other occupations	21.0% (6.4–50.9%)	Not serious[1]	Serious[17]	Serious[19]	Very serious[18]	Not assessed[7]	⊕◯◯◯ Very low
Environmental/mining/forestry workers	16.8% (6.2–38.3%)	Not serious[1]	Serious[17]	Not serious[3]	Very serious[18]	Not assessed[7]	⊕◯◯◯ Very low

GRADE certainty ratings: ⊕⊕⊕⊕, high certainty; ⊕⊕⊕◯ moderate certainty; ⊕⊕◯◯ low certainty; and ⊕◯◯◯ very low certainty. Because the evidence was derived from observational prevalence studies, the initial certainty was considered low. The certainty could not be downgraded below very low.

[1] Most included studies were classified as having moderate-to-high methodological quality according to the JBI Critical Appraisal Checklist, with no indication that methodological limitations substantially altered the pooled estimates.

[2] Downgraded by two levels because of considerable heterogeneity (I^2 = 96.7%), prevalence estimates ranging from 0% to 100%, and substantial variation across populations and settings.

[3] The populations, occupational exposures, serological outcomes, and settings were considered directly relevant to the review question.

[4] Downgraded because the confidence interval around the overall estimate was sufficiently wide to support meaningfully different interpretations of the magnitude of seroprevalence.

[5] Egger's regression test indicated statistically significant funnel plot asymmetry ($z = -2.79$, $p = 0.0053$). However, the negative direction indicated that smaller studies tended to report lower, rather than

higher, prevalence estimates, which is opposite to the conventional pattern associated with preferential publication of small studies reporting higher estimates. In addition, excluding the 11 studies in the lowest quartile of sample size reduced the pooled seroprevalence only modestly, from 18.3% (95% CI: 11.7–27.4%) to 16.3% (95% CI: 9.9–25.7%), supporting the robustness of the primary analysis. Because substantial heterogeneity may also produce funnel plot asymmetry, this finding was interpreted as evidence of potential small-study effects rather than definitive evidence of publication bias or inflation of the pooled prevalence.

[6] Considerable residual heterogeneity was observed among studies conducted in Asia (I^2 = 93.0%).

[7] Publication bias was not assessed separately within subgroups because subgroup-specific funnel plot and regression analyses were not performed and may have had insufficient statistical power.

[8] Considerable residual heterogeneity was observed among studies conducted in Africa (I^2 = 97.7%).

[9] Downgraded by two levels because of the very wide confidence interval, ranging from 9% to 58%.

[10] Considerable residual heterogeneity was observed among studies conducted in Oceania (I^2 = 95.7%).

[11] Downgraded because the confidence interval encompassed meaningfully different prevalence levels.

[12] Considerable residual heterogeneity was observed among studies conducted in the Americas (I^2 = 94.6%).

[13] Downgraded because substantial residual heterogeneity remained among studies conducted in Europe (I^2 = 72.8%).

[14] Downgraded by two levels because the estimate was based on only four studies and the confidence interval included values ranging from 0% to 9%.

[15] Considerable residual heterogeneity remained within all diagnostic subgroups (I^2 = 93.1%–96.5%).

[16] Downgraded because the estimate was based on a small number of studies and its confidence interval remained relatively wide.

[17] Downgraded because of clinical and methodological variability within the broad occupational categories.

[18] Downgraded by two levels because the confidence interval encompassed markedly different prevalence levels, limiting confidence in the magnitude of the estimate.

[19] Downgraded because the mixed/other category combined heterogeneous occupational populations and exposure pathways.
